# Early Cold-Induced Peroxidases and Aquaporins Are Associated With High Cold Tolerance in Dajiao (*Musa* spp. ‘Dajiao’)

**DOI:** 10.3389/fpls.2018.00282

**Published:** 2018-03-08

**Authors:** Wei-Di He, Jie Gao, Tong-Xin Dou, Xiu-Hong Shao, Fang-Cheng Bi, Ou Sheng, Gui-Ming Deng, Chun-Yu Li, Chun-Hua Hu, Ji-Hong Liu, Sheng Zhang, Qiao-Song Yang, Gan-Jun Yi

**Affiliations:** ^1^Key Laboratory of Horticultural Plant Biology of the Ministry of Education, College of Horticulture and Forestry Sciences, Huazhong Agricultural University, Wuhan, China; ^2^Key Laboratory of South Subtropical Fruit Biology and Genetic Resource Utilization of the Ministry of Agriculture/Guangdong Key Laboratory of Tropical and Subtropical Fruit Tree Research, Institute of Fruit Tree Research, Guangdong Academy of Agricultural Sciences, Guangzhou, China; ^3^Institute of Environmental Horticulture Research, Guangdong Academy of Agricultural Sciences, Guangzhou, China; ^4^College of Horticulture and Landscape, Hunan Agricultural University, Changsha, China; ^5^Institute of Biotechnology, Cornell University, Ithaca, NY, United States

**Keywords:** *Musa* spp. ‘Cavendish’, *Musa* spp. ‘Dajiao’, quantitative proteomics, cold tolerance, peroxidase, aquaporin

## Abstract

Banana is an important tropical fruit with high economic value. One of the main cultivars (‘Cavendish’) is susceptible to low temperatures, while another closely related specie (‘Dajiao’) has considerably higher cold tolerance. We previously reported that some membrane proteins appear to be involved in the cold tolerance of Dajiao bananas via an antioxidation mechanism. To investigate the early cold stress response of Dajiao, here we applied comparative membrane proteomics analysis for both cold-sensitive Cavendish and cold-tolerant Dajiao bananas subjected to cold stress at 10°C for 0, 3, and 6 h. A total of 2,333 and 1,834 proteins were identified in Cavendish and Dajiao, respectively. Subsequent bioinformatics analyses showed that 692 Cavendish proteins and 524 Dajiao proteins were predicted to be membrane proteins, of which 82 and 137 differentially abundant membrane proteins (DAMPs) were found in Cavendish and Dajiao, respectively. Interestingly, the number of DAMPs with increased abundance following 3 h of cold treatment in Dajiao (80) was seven times more than that in Cavendish (11). Gene ontology molecular function analysis of DAMPs for Cavendish and Dajiao indicated that they belong to eight categories including hydrolase activity, binding, transporter activity, antioxidant activity, etc., but the number in Dajiao is twice that in Cavendish. Strikingly, we found peroxidases (PODs) and aquaporins among the protein groups whose abundance was significantly increased after 3 h of cold treatment in Dajiao. Some of the PODs and aquaporins were verified by reverse-transcription PCR, multiple reaction monitoring, and green fluorescent protein-based subcellular localization analysis, demonstrating that the global membrane proteomics data are reliable. By combining the physiological and biochemical data, we found that membrane-bound Peroxidase 52 and Peroxidase P7, and aquaporins (MaPIP1;1, MaPIP1;2, MaPIP2;4, MaPIP2;6, MaTIP1;3) are mainly involved in decreased lipid peroxidation and maintaining leaf cell water potential, which appear to be the key cellular adaptations contributing to the cold tolerance of Dajiao. This membrane proteomics study provides new insights into cold stress tolerance mechanisms of banana, toward potential applications for ultimate genetic improvement of cold tolerance in banana.

## Introduction

Many of our existing staple crop species originating from tropical and subtropical regions are susceptible to damage when temperatures fall below 10°C, affecting growth, development, crop production, and geographical distribution of crop species (Lee et al., 2004). Plants are able to trigger a set of mechanisms to adapt to or survive exposure to non-freezing low temperatures gradually, which is termed cold acclimation (Guy, 1990). However, various species exhibit different adaptions to chilling stress, ranging from atrophy to death, even in different subclasses of the same species. Banana (*Musa* spp.) is one of the most productive fruits in the world, and the most important grain crop following rice, wheat, and maize in many developing countries. It originates in the tropics, and its growth is completely arrested and injured at 8°C. Since a large number of Cavendish bananas (*Musa* spp. ‘Cavendish’, AAA group) are grown in subtropical areas, such as China, Australia, Brazil, and Israel, cold spells often occur during winter or early spring leading to chilling injury. The extent of chilling injury is dependent on the intensity of cold exposure (temperature and duration). In severe cases, it results in damage to the whole plantation, but in mild cases, cold exposure inhibits fruit ripening and reduces the sensitivity of bananas to exogenous ethylene resulting in unpalatable fruits (Zhang et al., 2010; Hong et al., 2015; Elitzur et al., 2016). Compared with Cavendish, mature Dajiao (*Musa* spp. ‘Dajiao’, ABB group) shows considerably better adaptability to cold environments, enabling it to tolerate temperatures of 0–4°C. Therefore, Dajiao has been used as an excellent test case for investigation of cold-tolerance mechanisms in banana (Yang et al., 2012, 2015).

To understand the cold-resistance mechanisms of Cavendish and Dajiao, a large number of previous studies focused on physiological and biochemical aspects. For example, external application of hydrogen peroxide, calcium ions, salicylic acid, brassinolide, or methyl jasmonic acid was found to improve the cold resistance of cold-sensitive banana seedlings alleviating the resulting damage (Kang et al., 2003; Liu et al., 2008; Feng D. et al., 2009). In the past decade, a few genetic studies mainly on MAPK cascades-ICE1-CBF pathway and ABA signaling have been reported. Recently, comprehensive transcriptomic analyses have shown that banana PYL-PP2C-SnRK2 genes, the core components of ABA signaling, are involved in response to cold stress (Hu et al., 2017b) and MaMEKK1-MaMKK2 genes are activated by cold stress in cold-resistant banana (Wang et al., 2017). The rapid activation and selective induction of *ICE1* and *MYBS3* cold tolerance pathways in Dajiao may be one of the main reasons that Dajiao has higher cold resistance than Cavendish (Yang et al., 2015). *MpMYBS3*, as a crucial transcription factor of cold signaling, was found to confer cold tolerance in cold-sensitive banana (Dou et al., 2016). However, a limited number of studies have reported the role of membrane genes in banana cold resistance. A cold-resistance-related Dajiao membrane gene, *MpRCI*, has been identified, enhancing low-temperature resistance when heterologously expressed in transgenic tobacco (Feng D.-R. et al., 2009). Since poor correlation is often found between gene and protein expression levels in organisms, including under cold stress, proteomics is recognized as a good complement to genetic studies (Ning et al., 2012; Feussner and Polle, 2015). High-throughput proteomic profiles provide an effective approach to identify targeted candidate proteins in response to cold stress in some species (Long et al., 2012; Tian et al., 2015). A workflow for evaluating the membrane proteome of banana, a poorly sequenced plant, was proposed in Vertommen et al. (2011). Feng et al. (2015) reported that an increased antioxidation ability could represent a common characteristic of banana and plantain under cold stress conditions. Yang et al. (2012) identified some membrane-associated proteins and antioxidation mechanisms that contribute to the increased cold tolerance of Dajiao, indicating that MPs might also be important players in the cold response of Dajiao. It is well known that lower temperatures induce rigidification of membranes, leading to a disturbance of all membrane processes (e.g., opening of ion channels, membrane associated electron transfer reactions, etc.), and proteins associated with these membranes are suspected to play an important role in plant cold-tolerance. In the herbaceous monocot rice (*Oryza sativa*), which also originated in the tropics, overexpression of *COLD1^jap^*, a gene encoding a MP, significantly enhances chilling tolerance, whereas rice lines with a deficiency or down-regulation of *COLD1^jap^* become sensitive to cold stress (Ma et al., 2015). Driven by this knowledge, we sought to investigate the membrane proteomic differences between cold-sensitive Cavendish and cold-tolerant Dajiao.

For plant proteomics, the greatest challenges are to effectively reduce sample complexity and increase the effectiveness of proteomic analysis for confident identification of new, low-abundance proteins through MS (Fang and Zhang, 2008). Identification of low-abundance proteins in green tissue that contains high amounts of polyphenols and many highly abundant proteins is one of the main challenges in banana membrane proteomics. Reduction in the level of high-abundance proteins in extracts prior to MS analysis has proved an efficient way to improve detection of low-abundance proteins (Baracat et al., 2012). In addition, as a powerful tool for shotgun proteomics, isobaric tags for relative and absolute quantification (iTRAQ) have been widely applied in plant proteome analysis (Kosová et al., 2011). Due to its compatibility with two-dimensional LC for reducing sample complexity, the iTRAQ-based shotgun approach greatly facilitates the generation of in-depth global proteomic profiles and membrane proteomic profiles (Ross et al., 2004; Chen et al., 2010). Here, we used two steps of protein extraction and iTRAQ-based membrane proteome profiles to study the cold responses of the two most common banana genotypes.

Our group has a long-standing research interest in understanding the mechanisms of cold tolerance in *Musa* spp. Our previous global proteomics results suggested that some membrane or membrane-associated proteins are likely involved in cold tolerance of Dajiao via an antioxidation mechanism (Yang et al., 2012). Oxidative toxicity and dehydration are two common cellular reactions to chilling injury in plants. This study aimed to further investigate some suspected key MPs related to the cold stress response in Cavendish and Dajiao seedlings by iTRAQ-based comparative membrane proteomic analysis. Some of the important candidate MPs were assessed by RT-PCR analyses, verified by MRM-based targeted quantitation, and validated by additional enzyme activity assays.

## Materials and Methods

### Experimental Design and Proteomics Workflow

**FIGURE 1:**
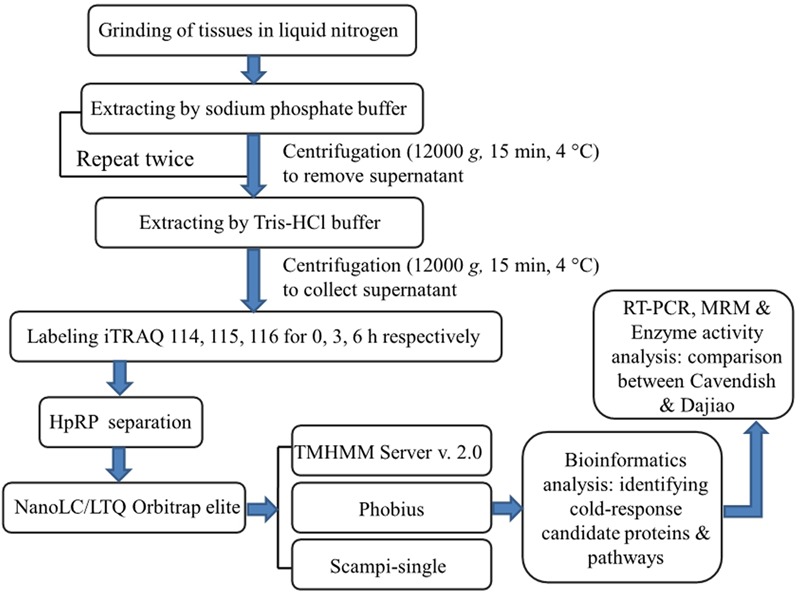
Experimental design and schematic diagram of the workflow used in this study. A two-step method was used for extraction of membrane proteins in Cavendish and Dajiao seedlings in response to cold stress (10°C for 0, 3, and 6 h), and three sets of biological replicate samples were analyzed by an iTRAQ-based 2D-LC/MS/MS workflow for examining proteome changes. Phobius, Scampi-single, and TMHMM programs were used for filtering and predicting membrane proteins. Some important candidate proteins identified were evaluated and verified by RT-PCR, MRM, recombinant green fluorescent protein (GFP)-based subcellular localization, and enzyme activity analyses.

The objective of this study was to apply comparative analysis of membrane proteomes between Cavendish and Dajiao for identification of potential cold-responsive membrane or membrane-associated proteins. The experimental design was based on the fact that Cavendish banana is sensitive to cold stress, while Dajiao has more cold tolerance than Cavendish. In this study, we focused on the early stage of cold stress (0, 3, and 6 h) for Cavendish and Dajiao based on the physiological and biochemical data we acquired. Given some inherent characteristics of MPs, which often pose big technical challenges to membrane proteomic research, we implemented an effective method in this study as shown in **Figure 1**. The main workflow covered (i) a two-step extraction method for enrichment of membrane or membrane-associated proteins; (ii) iTRAQ labeling and 2D LC-MS/MS for data acquisition; (iii) bioinformatics analysis for identifying changes in abundance of cold-responsive MPs; and (iv) RT-PCR, MRM target quantitation, and selective enzyme activity assays for data validation of DAMPs found in this study.

### Plant Materials and Cold-Stress Treatment

Seedlings of the cold-tolerant Dajiao (*Musa* spp. ‘Dajiao’, ABB group) and the cold-sensitive Cavendish (*Musa* spp. ‘Cavendish’, AAA group) with a uniform growth stage were obtained from the Institute of Fruit Tree Research, Guangdong Academy of Agricultural Sciences, Guangzhou, China. Seedlings were grown in a growth chamber under 30/28°C (day/night), a photon flux density of 240 μmol m^-2^ s^-1^ throughout a 12 h photoperiod, and a relative humidity of 60–80%. Six-leaf-stage seedlings were used in the experiment. Low temperature treatments started at 12:00 AM on the first day by setting the temperature to 10°C, which was reached about 40 min later. The first leaf from the top of each of the five plants was detached at each time point (0, 3, and 6 h at 10°C) for each biological replicate. The leaves from the five plants were cut into pieces (1.5 × 1.5 cm) and mixed well. Aliquots of the mixed tissues were frozen in liquid N_2_ and stored at -80°C until use. All samples from each time point were collected in triplicate.

### Physiological Measurement, Peroxidase Activity Assay, and Histochemical Staining

To test cold tolerance, seedlings of Cavendish and Dajiao were exposed to 10°C for 0, 3, 6, 24, and 48 h. Electrolyte leakage and MDA content were quantified using the relevant detection kits (Nanjing Jiancheng Bioengineering Institute, China) based on the manufacturer’s instructions. The soluble POD and ionically bound CWP enzyme activities were assayed according to the methods of Lin and Kao (2001) with minor modifications. Cavendish and Dajiao leaves (0.5 g) treated as described above were ground in an ice-cold mortar and pestle in 5 mL chilled 0.1 M phosphate buffer (pH 7.5) containing 1% (w/v) polyvinylpolypyrrolidone (PVP). The homogenates were centrifuged at 3,000 *g* for 15 min at 4°C. The supernatant was collected and used for assay of soluble POD activity. The resulting pellet was resuspended in the same buffer and centrifuged again (3,000 *g* for 15 min at 4°C). This washing procedure was repeated six times until negligible POD activity in supernatant from the final wash was detected. Finally, the washed pellet was incubated in 1 M sodium chloride for 2 h with shaking at 30°C and centrifuged at 3,000 *g* for 15 min at 4°C. The supernatant was used for ionically bound CWP enzyme activity assays. POD activity was determined with guaiacol at 436 nm. Protein concentrations were determined using the Bradford assay (Bradford, 1976) with bovine serum albumin (BSA) as a standard. One activity unit (U) was defined as an increase in absorbance at 234 nm of 0.01 unit min^-1^, and the results were expressed as relative activity. The activity results in each group were analyzed by Duncan’s test at the 95% confidence interval (*p* ≤ 0.05). Statistical analyses were performed with SPSS16.0 (SPSS, Chicago, IL, United States). The method for H_2_O_2_ and O_2_^∙-^ localization *in situ* was conducted as reported previously (Zhang et al., 2011).

To determine the effect of NaN_3_ on the activities of endogenous POD and CAT, six-leaf-stage seedlings were hydroponically grown in inhibitor-containing solutions (5 mM NaN_3_ for 12 h), using water as a control, before cold treatment at 10°C for 24 h, followed by analysis of H_2_O_2_ accumulation. Cavendish and Dajiao leaves were harvested after NaN_3_ treatment for activity assays using the relevant detection kits (Nanjing Jiancheng Bioengineering Institute, China) based on the manufacturer’s instructions.

### Subcellular Localization Analysis

To determine the subcellular localization of Peroxidase P7, the *Peroxidase P7* gene was amplified by PCR and cloned into the pYL322-d1 vector containing a GFP reporter gene (digested with *Eco*RI in advance) to produce the fusion construct pYL322-Peroxidase P7-GFP under control of the cauliflower mosaic virus (CaMV) 35S promoter by using a One Step Cloning Kit (Vazyme Biotech, Nanjing, China). The fusion construct and the control vector (pYL322-d1) were separately transferred into *Arabidopsis thaliana* protoplasts (Yoo et al., 2007) and Cavendish protoplasts (Sagi et al., 1994). The transformed protoplasts were visualized under a universal fluorescence microscope (Olympus BX61, Tokyo, Japan).

### Protein Extraction

A two-step method was conducted for the extraction of MPs from leaves (three biological replicates of Cavendish and Dajiao seedlings at 10°C for 0, 3, and 6 h) according to the method of Vertommen et al. (2011) with minor modifications. For the first step, leaf tissues were ground in liquid nitrogen using a pre-chilled mortar and pestle before sodium phosphate buffer (100 mM, pH 7.5) containing 1 mM ethylenediaminetetraacetic acid (EDTA), 100 μg mL^-1^ phenylmethylsulfonyl fluoride (PMSF), and 2% (w/v) poly(vinylpolypyrrolidone) (PVPP) was added to the ground tissues (4 mL buffer g^-1^ tissues). The homogenate was centrifuged at 12,000 *g* at 4°C for 15 min. The supernatant was removed, and an equal volume of sodium phosphate buffer was added to resuspend the protein pellet. After vortexing on ice for 3 min, the homogenate was centrifuged at 12,000 *g* at 4°C for 15 min and the resulting supernatant was discarded. Finally, the proteins collected in the pellet were resuspended using the above steps twice. For the second step, the protein pellet from the first step was ground using an ice-cold mortar and pestle with Tris-HCl buffer according to the method of Sadowski et al. (2006) with minor modifications [0.1 M Tris-HCl, pH 8.3, 5 M Urea, 2 M Thiourea, 2% (w/v) 3-(3-Cholamidopropyl)dimethylammonio-propanesulfonic acid (CHAPS), 50 mM DL-dithiothreitol (DTT), 0.1% (w/v) sodium dodecyl sulfate (SDS), 0.5% (v/v) Pharmalytes 3–10, 100 μg mL^-1^ PMSF]. The homogenate was centrifuged at 12,000 *g* for 15 min, and the supernatant was collected for subsequent protein quantitation analysis.

### Protein iTRAQ Labeling, High pH Reverse Phase (hpRP) Fractionation, nanoLC-MS/MS Analysis, and Database Search

The methods and processes of protein quantitation, iTRAQ labeling, first-dimensional LC fractionation by hpRP separation, and nanoLC-MS/MS analysis were carried out as described by Deng et al. (2015). Briefly, the Dajiao samples were labeled with iTRAQ tag-114, tag-115, and tag-116 for cold treatment at 0, 3, and 6 h, respectively. Each biological replicate of Dajiao samples was labeled in a separate set. The same labeling design was used for the three sets of Cavendish samples. After a labeling check, the three labeled samples comprising each of the six sets were combined and subjected to hpRP fractionation into 12 fractions that were analyzed by nanoLC-MS/MS on an LTQ-Orbitrap Velos mass spectrometer (Thermo-Fisher Scientific, San Jose, CA, United States). The following modifications were applied: fixed MMTS modification of cysteine; variable modification for deamidation of Asn and Gln residues; 4-plex iTRAQ modifications on Tyr, Lys, and N-terminal amines; and variable modifications of methionine oxidation. The peptide and fragment mass tolerance values were 20 ppm and 0.1 Da, respectively. The target-decoy strategy was used to estimate the FDR in each replicate set according to the study of Elias and Gygi (2007). FDR less than 2% was counted as acceptable. To reduce the probability of false peptide identification, only peptides with significance scores ≥ 20 at the 99% confidence interval by a Mascot probability analysis of greater than “identity” were used. Furthermore, it was required that each confident protein identification involve at least two unique peptide identifications indicated in Mascot, confidently quantified in subsequent evaluation.

All raw spectra files were processed using Proteome Discoverer 1.4 (PD1.4, Thermo), and the spectra from each DDA file were output as an MGF file for subsequent database search using in-house-licensed Mascot Daemon (version 2.3.02, Matrix Science, Boston, MA, United States) against a banana protein database containing 36,538 entries downloaded from the following website: http://banana-genome-hub.southgreen.fr/download. Proteins were identified by searches using the transmembrane structure prediction software tools TMHMM Server v. 2.0 (Krogh et al., 2001), Phobius (Reynolds et al., 2008), and Scampi-single (Bernsel et al., 2008) according to the methods of Nam et al. (2009). Only candidate proteins that were predicted by two out of the three software programs were deemed to be MPs. The change in relative concentration of any given protein after 3 and 6 h of treatment, in Cavendish and Dajiao, was obtained from the iTRAQ 4-plex reporter ion ratios (115/114 and 116/114, respectively) based on a weighted average of all confidently identified peptides. Proteins identified and quantified in all three sets of Cavendish or Dajiao samples were used to statistically assess the quantitative variations from biological and analytical triplicates. The internal error plots for assessment of the biological replicate variation were used to determine the cutoff ratio. Additionally, a two-sample student *t*-test between the two groups was carried out, and a *p*-value was obtained for each of the quantified proteins. As a result, the functional annotation and classification of all MPs exhibiting ± 1.4-fold change in abundance at 3 and 6 h compared with the 0 h control and a *p*-value ≤ 0.05 were analyzed using Blast2go (Bioinformatics Department, CIPF, Valencia, Spain) (Conesa and Götz, 2007; Götz et al., 2008).

### Quantitative RT-PCR and MRM Analyses

Cavendish and Dajiao leaf samples in three biological replicates (treated at 10°C for 0, 3, 6, 24, and 48 h) were collected for total RNA isolation. The first-strand cDNA was synthesized according to the Prime Script^TM^ RT-PCR kit protocol (Takara, Japan). Primer pairs for RT-PCR were designed using Primer Premier 5.0 (Premier Biosoft, Palo Alto, CA, United States). PCR reactions were performed on a LightCycler480^®^ II with LightCycler480 Service Software (Roche, Germany) by using the LightCycle^®^ Premix EX Taq (Perfect Real Time) Kit protocol (Takara, Japan). The efficiency of primers for the target gene and internal control gene (*25S ribosomal RNA*) was assessed. No-template controls were also set for each primer pair as a blank control.

The Skyline 3.1 program was used to generate an initial MRM transition pair list for three selected DAMPs including two peroxidases and an aquaporin, as described previously (Martin et al., 2016). Filters for transition settings in MRM analysis were set up for three peptides per protein, and three transition pairs per peptide, and exported directly from Skyline 3.1 into an excel file that was imported into Analyst 1.6 software in the 4000 QTRAP. The MRM-IDA runs were used to optimize the selection of the Q3 product ions in transition ion pairs. The final transition ion pair list was further refined based on the MRM-IDA results. The MRM data for the Cavendish and Dajiao triplicate samples were analyzed using MultiQuant 2.2 software (SCIEX, Framingham, MA, United States). The mean and SD of the percentage abundance of each peptide were determined from the triplicate runs of each sample.

## Results

### Physiological and Biochemical Responses of Cavendish and Dajiao to Cold Stress

**FIGURE 2:**
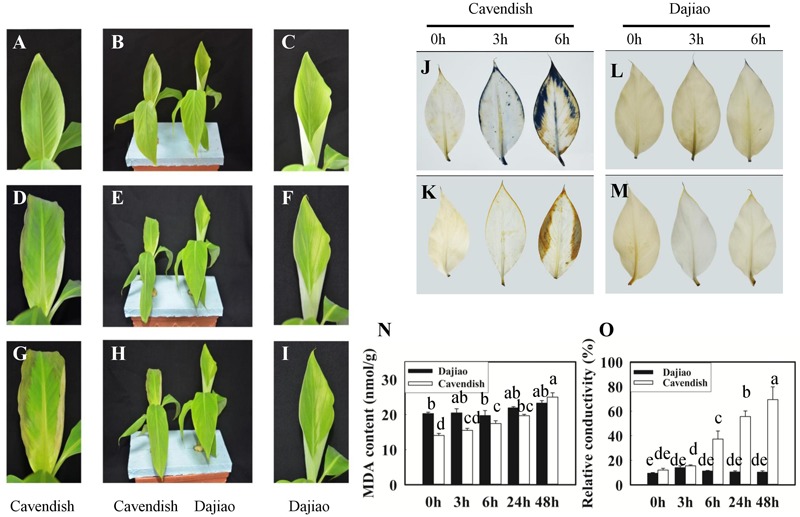
Phenotype, reactive oxygen species (ROS) accumulation, malondialdehyde (MDA) content, and relative conductivity in Cavendish and Dajiao under cold stress. Six-leaf-stage seedlings of Cavendish and Dajiao were treated at 10°C for 0, 3, and 6 h (**B,E,H**, respectively), and one representative leaf is shown, respectively **(A,C,D,F,G,I)**. The level of superoxide radicals and H_2_O_2_ in the leaves of Cavendish and Dajiao was assessed by NBT **(J,L)** and DAB **(K,M)** staining, respectively. MDA content **(N)** and relative conductivity **(O)** in the leaves of Cavendish and Dajiao were measured under treatment at 10°C for 0, 3, 6, 24, and 48 h. Data are the mean ± SE (*n* = 5). Different letters indicate significant differences at *p* ≤ 0.05 between control and treatment.

The exposure of Cavendish and Dajiao seedlings to low temperature induced many changes in physiological and biochemical parameters. After cold treatment for 3 and 6 h at 10°C, Cavendish leaves displayed slight wilt that developed gradually into observed necrotic spots, while Dajiao leaves remained unchanged (**Figures 2A–I**). We also found that high levels of ROS accumulated in the cell when plants were exposed to low temperature. **Figures 2J–M** shows the level of superoxide radicals and H_2_O_2_ in the leaves of Cavendish and Dajiao with NBT and DAB staining, respectively. For superoxide radical detection, blue formazan deposits represent the characteristic reaction of NBT with superoxide radical. The blue deposits were increased in the leaves of both Cavendish and Dajiao with increased low temperature exposure time (**Figures 2J,L**). However, nearly one third of the Cavendish leaf area was stained with blue deposits after treatment at 10°C for 6 h, while only a small part of the leaf edge was stained in Dajiao. For H_2_O_2_ detection, brown deposits are the result of the reaction of DAB with H_2_O_2_. The result was quite similar to that for superoxide radical detection, and Cavendish leaves were stained more intensely than Dajiao leaves (**Figures 2K,M**). To investigate damage to the cell membrane in Cavendish and Dajiao seedlings under low temperature, seedlings were exposed to 10°C for 0, 3, 6, 24, and 48 h. Significantly increased MDA content (**Figure 2N**) and cell membrane permeability (**Figure 2O**) were observed in Cavendish after 6 h, but no equivalent changes were seen in Dajiao even at 48 h.

### Detection of Cold-Stress-Induced Cavendish and Dajiao Membrane Proteins

**FIGURE 3:**
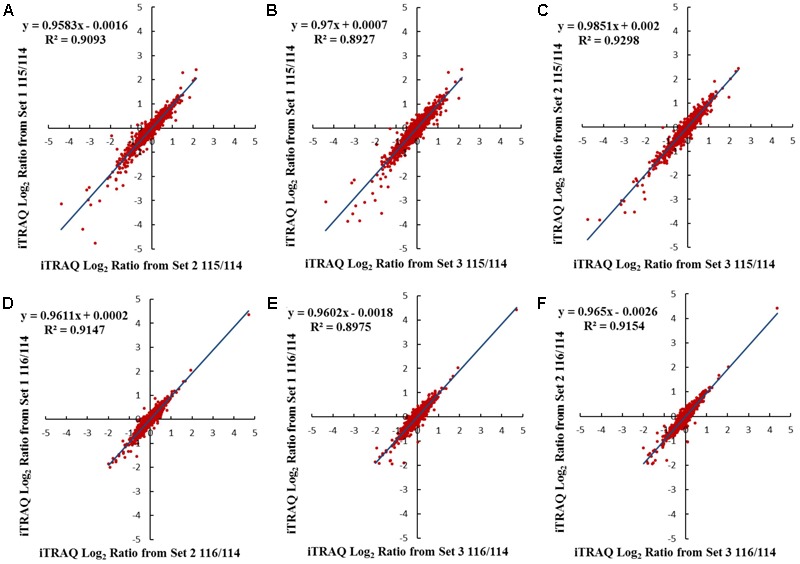
Comparison of log_2_ iTRAQ ratio (115/114 and 116/114) for 1,834 proteins identified in all three Dajiao biological replicates: set1, set2, and set3. Plots of 115/114 or 116/114 ratios for each of the quantified proteins between two of the three sets generated comparable quantification results, as determined by a linear regression analysis that revealed a slope of around 0.96, 0.97, or 0.99 in Dajiao for 115/114, among the pairs of set1/set2 **(A)**, set1/set3 **(B)**, and set2/set3 **(C)**, respectively. Similar plots were also conducted for the 116/114 ratio, yielding a slope of around 0.96, 0.96, or 0.97 in Dajiao, among the pairs of set1/set2 **(D)**, set1/set3 **(E)**, and set2/set3 **(F)**, respectively.

iTRAQ-based membrane proteomics analysis was conducted to investigate the proteome profiles of Cavendish and Dajiao under cold stress for 3 and 6 h compared with the 0 h control, respectively (115/114 and 116/114). The results of protein identification and quantitation from three independent biological replicates are listed in **Supplementary Data S1**. Comparison of results for the three sets of biological triplicate samples from both Cavendish and Dajiao are presented as Venn diagrams. A total of 5,246 and 4,282 distinct proteins in Cavendish and Dajiao, respectively, were identified with an estimated 2% FDR, of which 3,487 (66%) and 2,836 (66%) overlapped in all three replicate data sets of Cavendish and Dajiao, respectively (**Supplementary Figure S1**). Out of the total number of identified proteins, 3,895 and 3,071 proteins were confidently quantified with at least two peptides hit per protein, of which 2,333 (60%) and 1,834 (60%) were commonly identified in all three sets (**Supplementary Figure S2**), respectively. As shown in **Figure 3** and **Supplementary Figure S3**, plots of 115/114 or 116/114 ratios for each of the quantified proteins between two of the three sets generated comparable quantification results, as determined by a linear regression analysis that revealed a slope of around 0.88, 0.87, or 0.87 in Cavendish and 0.96, 0.97, or 0.99 in Dajiao for 115/114, among the pairs of set1/set2, set1/set3, and set2/set3, respectively. Similar plots were also conducted for the 116/114 ratio, yielding a slope of around 0.88, 0.87, or 0.89 in Cavendish and 0.96, 0.96, or 0.97 in Dajiao. Phobius, Scampi-single, and TMHMM were used as a consensus approach for filtering, and, as a result, 692 and 524 MPs were identified in Cavendish and Dajiao, which represents 29.7 and 28.6% of the quantified proteins reported above, respectively. The average internal errors for the three plots at log_2_ scale were 0.48 in both Cavendish and Dajiao, which corresponds to 1.40-fold internal variation. The average deviation of ±0.48 at log_2_ along with a *p*-value ≤ 0.05 was then used as a significance threshold in determining DAMPs. All DAMPs are listed in **Supplementary Data S1**.

Combining membrane proteomics data from both Cavendish and Dajiao, a total of 191 DAMPs were identified, 54 DAMPs were found exclusively in Cavendish, 109 DAMPs in Dajiao only, and 28 DAMPs in both. The detailed results are shown in **Table 1**. A total of 11 and 43 MPs with increased abundance were found in Cavendish following 3 and 6 h of cold treatment, respectively, while 80 and 32 MPs with increased abundance were detected in Dajiao following 3 and 6 h of cold stress, respectively. Meanwhile, 29 and 14 MPs in Cavendish, and 33 and 28 MPs in Dajiao were decreased in abundance after 3 and 6 h of cold stress, respectively. There were three and 13 MPs with increased abundance that were commonly detected in both Cavendish and Dajiao at 3 and 6 h of cold treatment, respectively. Candidate proteins with decreased abundance found in both genotypes of banana numbered 6 and 13 at 3 and 6 h of cold treatment, respectively. Overall, the percentage of DAMPs that were commonly identified in both Cavendish and Dajiao was considerably low (3∼21%) under cold stress. Our data also show that 40 and 57 DAMPs were identified for 3 and 6 h of cold stress, respectively, in Cavendish, while 113 and 60 DAMPs were identified for 3 and 6 h of cold stress, respectively, in Dajiao.

**Table 1:** Summary of differentially abundant membrane proteins (DAMPs) in Cavendish and Dajiao under cold stress for 3 and 6 h.

		Cavendish (unique only)	Dajiao (unique only)	Common to both
DAMPs at 3 h	Total	40 (31)	113 (104)	9 (6%)
	Up-regulated (≥1.4)	11 (8)	80 (77)	3 (3%)
	Down-regulated (≤0.714)	29 (23)	33 (27)	6 (11%)
DAMPs at 6 h	Total	57 (41)	60 (46)	16 (16%)
	Up-regulated (≥1.4)	43 (30)	32 (24)	13 (21%)
	Down-regulated (≤0.714)	14 (11)	28 (22)	3 (8%)

**FIGURE 4:**
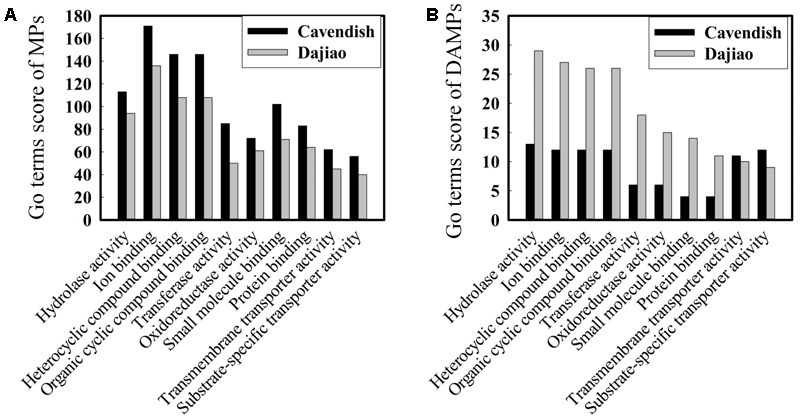
Comparison of GO molecular functions for Cavendish and Dajiao MPs **(A)** and DAMPs **(B)** under cold stress.

Blast2go, a GO annotations based protein-grouping software, was used to describe functions of the identified DAMPs. **Figure 4A** shows that there was no difference in distribution of molecular function categories for the MPs between Cavendish and Dajiao. However, the number of DAMPs in Dajiao was more than double in eight of ten categories compared with those in Cavendish (**Figure 4B**). These eight functional categories included hydrolase activity, ion binding, heterocyclic compound binding, organic cyclic compound binding, transferase activity, oxidoreductase activity, small molecule binding, and protein binding. The number of MPs in both Cavendish and Dajiao showed similar results for all biological process categories (**Supplementary Figure S4**). Consistent with the findings in molecular function categories, the number of DAMPs in Dajiao was nearly double that in Cavendish for five biological process categories (organic substance metabolic process, primary metabolic process, cellular metabolic process, single-organism metabolic process, and response to stress). Pathway enrichment analysis was performed through Kyoto Encyclopedia of Genes and Genomes (KEGG) with association signals at the *p* ≤ 0.05 level, and a total of 15 and 30 pathways were found to be significantly enriched in Cavendish and Dajiao, respectively (**Supplementary Figure S5**). These pathways encompassed oxidative phosphorylation, purine metabolism, phenylalanine metabolism, starch and sucrose metabolism, galactose metabolism, etc.

### Peroxidase P7 Is Localized in the Plasma Membrane and Chloroplast

**FIGURE 5:**
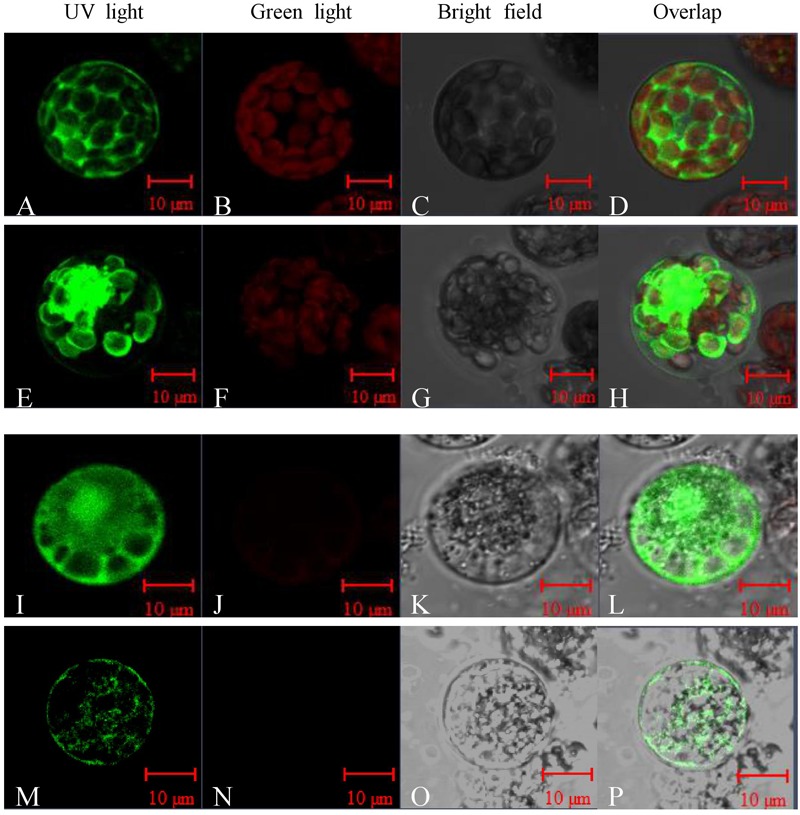
Subcellular localization analysis of Peroxidase P7. The Peroxidase P7-GFP fusion protein and GFP (used as a control), driven by the CaMV 35S promoter, were separately transformed into *Arabidopsis thaliana* protoplasts **(A–H)** and Cavendish protoplasts **(I–P)** and visualized by fluorescence microscopy. Images were taken of the representative cells expressing GFP or Peroxidase P7-GFP fusion protein under UV light **(A,E,I,M)**, green light **(B,F,J,N)**, or bright field **(C,G,K,O)**. The merged images are shown in **(D,H,L,P)** respectively.

Sequence analysis showed that Peroxidase P7 possesses a plasma membrane and chloroplast localization signal. To test this, the Peroxidase P7*-*GFP fusion protein and GFP (used as a control), driven by the CaMV 35S promoter, were separately transformed into *Arabidopsis thaliana* protoplasts and Cavendish protoplasts (**Figure 5**), and visualized by fluorescence microscopy. Transient expression assays in *Arabidopsis thaliana* (**Figures 5A–D**) and Cavendish (**Figures 5I–L**) protoplasts indicated that GFP alone was detected in the plasma membrane, cytoplasm, and nucleus, whereas the Peroxidase P7-GFP fusion protein was detected in the plasma membrane and chloroplasts (**Figures 5E–H**). These observations suggested that the subcellular location of Peroxidase P7 is the plasma membrane and chloroplast. Plasma membrane and plastid localization of Peroxidase P7 was further confirmed by transformation of Cavendish protoplasts (**Figures 5M–P**).

### Confirmation of Cold-Induced Candidate Membrane Proteins by Quantitative RT-PCR

**FIGURE 6:**
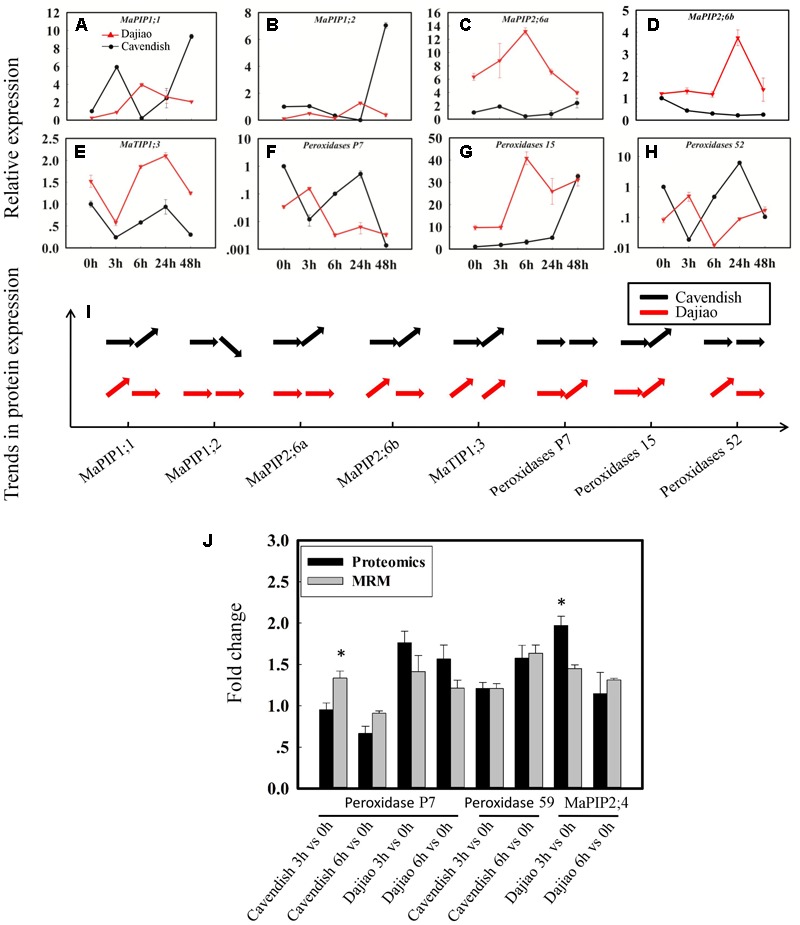
Time-course expression and MRM analysis of representative differentially abundant proteins under cold stress. Relative expression of five aquaporins **(A–E)** and three peroxidases **(F–H)**, and their expression patterns in proteomics data **(I)**. The horizontal arrow, upwardly tilted arrow, and downwardly tilted arrow represent no change, and increased and decreased expression, respectively. Relative quantitation of representative DAMPs between Cavendish and Dajiao by MRM analysis **(J)**. The MRM data were obtained by summing the six or nine peak areas, generated from three biological replicates, for each protein containing two or three typical peptides, along with three transition ion pairs for each targeted peptide. Asterisks indicate significant differences between the transgenic lines and wild type under the same conditions (^∗^*p* ≤ 0.05).

In validation of quantitation results from global proteomics analysis, seven aquaporins, four peroxidases, two glucan endo-1,3-beta-glucosidases, two GDPDs (glycerophosphoryl diester phosphodiesterases), four inactive receptor kinases At2g26730, an ABC transporter A family member 7, a Nicotiana lesion-inducing protein, and seven aspartic proteinases were selected as candidate MPs possibly associated with the high cold tolerance in Dajiao. Those representative DAMPs under cold stress were confirmed by quantitative RT-PCR (protein accessions and primers are shown in **Supplementary Data S2**). According to our proteomics data, MaPIP1;1 and MaPIP2;6 were cold-induced earlier in Dajiao than in Cavendish, while MaPIP1;2 was down-regulated in Cavendish at 6 h but no change was seen in Dajiao (**Supplementary Table S1**). According to RT-PCR data, *MaPIP1;1* and *MaPIP2;6a* were up-regulated in Dajiao at 6 h but down-regulated in Cavendish, while *MaPIP1;2* and *MaPIP2;6b* were up-regulated in Dajiao at 3 h but showed either no change or down-regulation in Cavendish (**Figures 6A–D**). Time-course expression analysis of *Peroxidase 15, Peroxidase P7*, and *Peroxidase 52* is shown in **Figures 6F–H**. The expression pattern of *Peroxidase 15* in both RT-PCR and proteomics data was the same between Cavendish and Dajiao, being significantly up-regulated at 6 h in both (**Figures 6G,I**). Both *Peroxidase P7* and *Peroxidase 52* shared the same expression pattern in RT-PCR; they were up-regulated in Dajiao and down-regulated in Cavendish at 3 h (**Figures 6F,H**). Meanwhile, there was no change in expression of Peroxidase P7 or Peroxidase 52 in Cavendish but increased expression in Dajiao under cold treatment in our proteomics data (**Figure 6I** and **Supplementary Table S2**). As shown in **Supplementary Figures S6A–H**, seven of the eight representative genes were increased in abundance at 3 or 6 h followed by a downward trend in Dajiao. By contrast, there was less obvious increase in expression in Cavendish during the first 6 h, which was consistently in line with proteomics data (**Supplementary Figure S6I**).

### Confirmation of Cold-Induced Candidate MPs by MRM

To cross-validate the identity of the cold-responsive MPs, we initially used immunoblot analysis to assess the expression of ten selected DAMPs as determined by iTRAQ. Unfortunately, the experiments were not successful because none of the antibodies were specific enough. Thus, we decided to use target-based peptide quantitation (MRM) for validation of cold-induced candidate MPs. Peroxidase P7, Peroxidase 59, and MaPIP2;4 were chosen as representative proteins for detection by MRM (**Figure 6J**). The results from MRM analyses were generally in good agreement with the proteomics data for all of these three proteins.

### Inhibition of Peroxidase Activity Compromises Cold Tolerance of Dajiao

**FIGURE 7:**
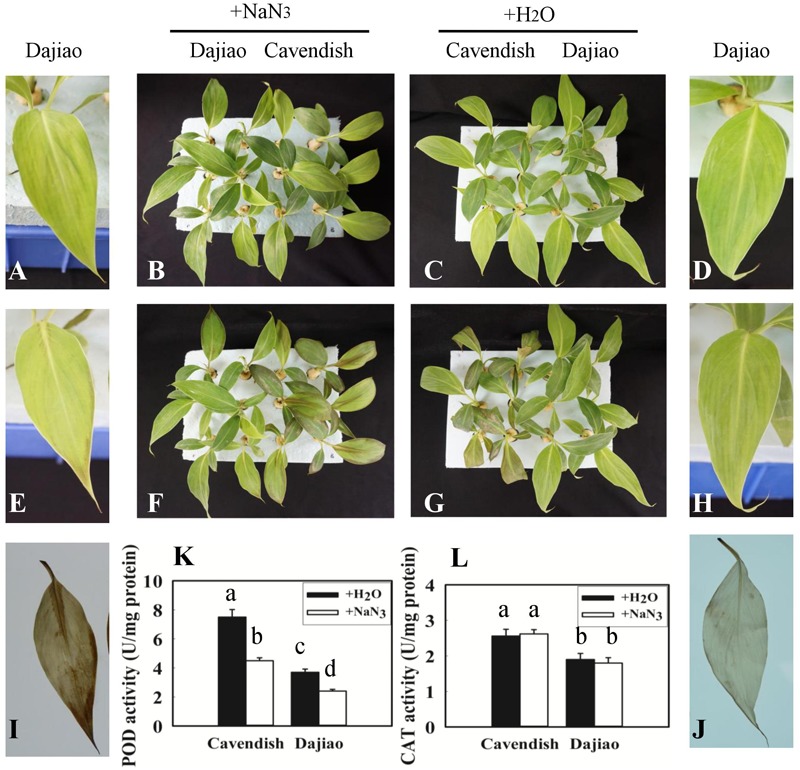
Treatment with NaN_3_ compromised cold tolerance of Dajiao. Phenotype of the Cavendish and Dajiao plants pretreated with NaN_3_ **(B)** or water **(C)**, followed by cold stress at 10°C for 24 h **(F,G)**; representative leaves of Dajiao are shown **(A,D,E,H)**. H_2_O_2_ accumulation **(I,J)** in leaves of Dajiao analyzed at the end of cold treatment. **(K,L)** Show the comparison of POD and CAT activities in Cavendish and Dajiao plants treated with water or 5 mM NaN_3_ for 12 h. Different lowercase letters above columns indicate a significant difference at *p* ≤ 0.05 between the columns by Duncan’s test using SPSS statistical software (version 16.0, SPSS Inc. Chicago, IL, United States).

After Cavendish and Dajiao seedlings were treated with NaN_3_, a specific peroxidase inhibitor (Zhan et al., 2003), no phenotypic difference was observed between seedlings treated with NaN_3_ and water under optimal temperature after 24 h (**Figures 7B,C**). However, phenotypic differences in Dajiao and Cavendish after treatment with water or NaN_3_ at 10°C for 24 h were found (**Figures 7F,G**). When the seedlings were treated with NaN_3_ and subsequently exposed to cold, both Dajiao and Cavendish seedlings showed remarkable damage as compared with the treatment with water. A similar result was observed by DAB staining for H_2_O_2_. When treated with NaN_3_, the seedlings of Dajiao showed more intense DAB staining compared with the treatment with water (**Figures 7I,J**). To determine how NaN_3_ affected endogenous antioxidant enzymes, the activities of peroxidase and CAT (two enzymes involved in H_2_O_2_ scavenging) were analyzed following NaN_3_ treatment. We found that treatment with NaN_3_ led to a significant reduction in peroxidase activity relative to water treatment (from 3.7 to 2.4 and 7.5 to 4.5 U mg^-1^ protein in Dajiao and Cavendish, respectively), whereas CAT activity was negligibly altered (**Figures 7K,L**).

**FIGURE 8:**
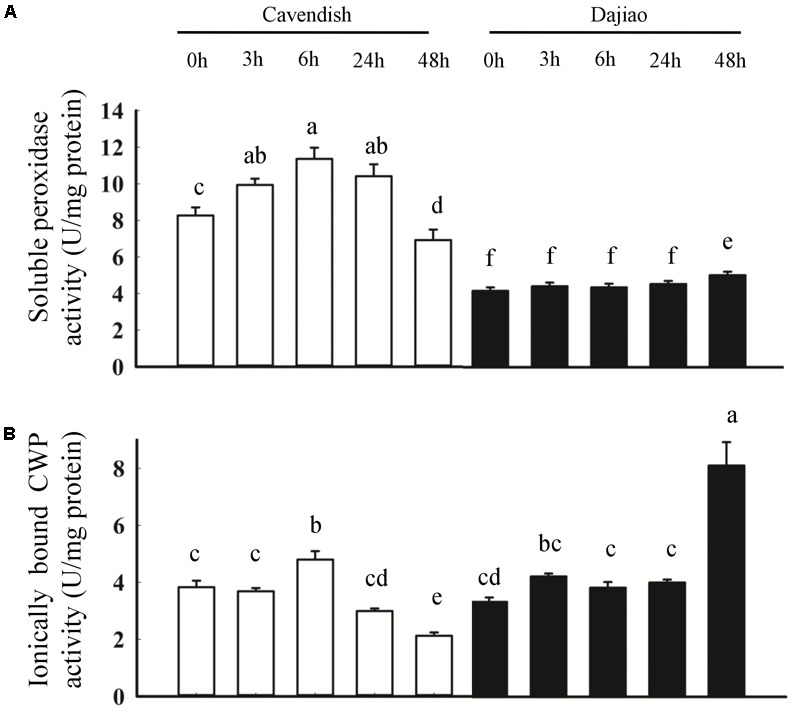
Peroxidase activity assays for Cavendish and Dajiao. Peroxidase activity assays reveal the temporal changes in soluble peroxidase activity **(A)** and ionically bound CWP activity **(B)** for both Cavendish and Dajiao under 10°C treatment for the indicated time. Values of relative activity for each column indicate means ± SD of three biological replicates. Different lowercase letters above columns indicate a significant difference at *p* ≤ 0.05 between the columns by Duncan’s test using SPSS statistical software (version 16.0, SPSS Inc. Chicago, IL, United States).

To further investigate the role of peroxidases in cold tolerance, soluble peroxidase activity and ionically bound CWP activity were determined (**Figure 8**). As shown in **Figure 8A**, the soluble peroxidase activity revealed a sustained increase at 3 and 6 h, followed by a decrease at 24 and 48 h in Cavendish, whereas it remained stable until a small increase at 48 h in Dajiao. In addition, the soluble peroxidase activity in Cavendish was almost twice that observed in Dajiao. The ionically bound CWP activity showed a different pattern compared with the soluble peroxidase activity, as shown in **Figure 8B**. In Cavendish, the CWP activity was induced at 6 h but remained at a lower level at 24 and 48 h, as at 0 h. However, the enzyme level in Dajiao was increased at 3 h and then remained stable until a second significant increase at 48 h. Unlike soluble POD activity, the overall CWP activity of Cavendish and Dajiao was at a similar level.

## Discussion

### Experimental Design and Proteomics Workflow

In general, MPs and membrane-associated proteins are able to effectively and/or transiently control some metabolic pathways to cope with abiotic stress, such as low temperature. MPs appear to play a key role in catalytic reactions, drug targets, communication, and organelle-organelle, organelle-cell, cell-cell and cell-environment interactions. Compared with analysis of other classes of proteins, membrane proteomics analysis has remained a challenge in large part due to the poor solubility of MPs in aqueous media and their low abundance. For a membrane proteomic study, obtaining high-concentration protein samples is essential. A workflow for membrane proteomics in banana, a poorly sequenced plant, was initially reported in Vertommen et al. (2011), but the MP extraction process is very complex and may affect the accuracy of proteome results. With the banana genome sequence being published (D’Hont et al., 2012), transmembrane structure prediction software tools can be readily used to predict MPs. Thus, it is not essential to separate MPs from the total proteins for identification of the MPs. The presence of soluble proteins and variable contaminants in the samples is generally the main limiting factor for obtaining high-quality MP samples. In this study, we focused on whole-cell MPs for global analysis. We developed a two-step protein extraction method, suitable for different hydrophobic proteins and efficient extraction of broad membrane or membrane-associated proteins from complex samples (Mark and David, 1998). First, highly abundant cytoplasmic proteins with relatively high hydrophilicity, which is expected to interfere with isolation and identification of MPs, were readily removed from the samples by using sodium phosphate buffer. Subsequently, a modified extraction buffer containing additional urea and thiourea in Tris-HCl buffer was optimized and used to extract MPs in this study. Protein yields from plant species are reported to range from 0.09 to 28.33 mg g^-1^ fresh weight (Amalraj et al., 2010; Dziedzic and Mcdonald, 2012) depending on the type of plant material and the extraction methods used. Following removal of most of the high-abundance proteins, the MP yield we obtained with the two-step method was about 0.82 mg g^-1^ fresh weight, suggesting that the modified extraction method we implemented works well.

Significantly increased MDA content (**Figure 2N**) and cell membrane permeability (**Figure 2O**) were observed in Cavendish after 6 h, but no equivalent changes were seen in Dajiao, even at 48 h, which suggests that the plasma membranes in Cavendish, but not those in Dajiao, suffer severe damage under cold treatment. According to these data, 6 h appears the observed decay time point for MPs to play adaptation role in cold resistance. Therefore, we chose 0, 3, and 6 h as the time points for membrane proteomic analysis. The reliability of our proteomics dataset was assessed in several aspects. First of all, the reproducibility of protein IDs and protein IDs with quantification ratios for Cavendish and Dajiao, counting all three sets, was 66% and 60%, respectively (**Supplementary Figures S1, S2**), indicating that Cavendish and Dajiao showed a similar and high qualitative reproducibility in membrane proteomics data. Second, in the quantitative precision and reproducibility assessment, the slope of the linear regression analysis for variance among the three sets of data ranged from 0.87 to 0.99, suggesting that the global quantitative iTRAQ data in triplicate were quite reproducible. Third, to improve prediction accuracy of MPs, the proteins containing at least one membrane region predicted by at least two of the three web programs (Phobius, Scampi-single, and TMHMM) were determined as MPs in this study. As a result, we recovered a 29% yield of the membrane proteome (524 MPs out of 1,834) for Dajiao and ∼30% yield for the Cavendish membrane proteome (692 out of 2,333), which is in the upper range of estimated percentage rates for all genes encoding MPs (Krogh et al., 2001), demonstrating again that our workflow for membrane proteomics works well. To verify the reliability of the transmembrane prediction method, Peroxidase P7 as a representative protein was confirmed to be a MP by GFP-based subcellular localization analysis in both *Arabidopsis* and Cavendish, as shown in **Figure 5**. Finally, a total of 16 representative DAMPs under cold stress were confirmed by quantitative RT-PCR along with three representative DAMPs being verified by MRM analysis, and almost all expression patterns were consistently in line with the proteomics data, indicating the reliability of our membrane proteomics datasets (**Figure 6** and **Supplementary Figure S6**).

Our previous transcriptome study showed that the number of up- and down-regulated genes in Cavendish (243) is more than that in Dajiao (71) (Yang et al., 2015). However, the number of DAMPs identified in this study was considerably different in Cavendish (82) and Dajiao (137) under the same cold treatment. The difference is not totally surprising given the following reasons with the different analysis techniques and workflows for obtaining each of the transcriptome and membrane proteome datasets. First of all, transcriptomic data was acquired for the total RNA extracted from the cold-treated leaf samples, while the membrane proteome data is a subset proteome that contains only ∼30% of the predicted global proteome. Second, a much broader dynamic range of transcripts is generally detected by RNA-Seq (with PCR amplification) in transcriptomics than that of their counterparts (proteins) detected by iTRAQ-based shotgun proteomics. Finally, a poor correlation between gene and protein expression levels (Ning et al., 2012) plus different data analysis programs (e.g., FDR and statistical parameters) with different cutoff ratios (e.g., twofold for transcripts and 1.4-fold for MPs) for determining differentially expressed transcripts/proteins would unavoidably contribute to the discrepancy. Despite the difference in the number of cold-response candidates found in the two datasets, some important molecular functions such as antioxidant activity did show consistent results as discussed below. Compared with the transcriptome, the global and subset proteomes can provide a wealth of unique information such as post-translational modifications and protein-protein interactions for cellular regulatory processes (Feussner and Polle, 2015). We believe the proteome is an important complement to the transcriptome.

In GO analysis, DAMPs were classified into two major categories: molecular function (MF) and biological process (BP). **Figure 4** and **Supplementary Figure S4** show that there was no difference in the distribution of MF and BP categories for the MPs and DAMPs between Cavendish and Dajiao, suggesting that cold stress appears to have a broad range of impacts on both banana genotypes. However, the number of DAMPs in Dajiao compared with those in Cavendish was more than double in eight of the ten MF and five of the ten BP categories. Moreover, the number of DAMPs up-regulated in Dajiao at 3 h (80), was seven times more than those (11) in Cavendish (**Table 1**). This observation supports the notion that the response to low temperature stress appears more readily adapted in cold-tolerant Dajiao than in cold-sensitive Cavendish. ROS signaling networks were found to be more active in FJ (cold-tolerant banana) than in Cavendish under cold treatments, which may contribute to the strong cold tolerance of FJ (Hu et al., 2017a). ROS accumulation and wilting with dehydration are physiological and biochemical phenotypes of Cavendish under cold treatment (**Figures 2A–M**). Therefore, we suspect that those proteins with an increased abundance at 3 h are involved in the early stage response under cold treatment and their pathways are likely to be critically involved in cold tolerance of Dajiao.

The most striking findings in our proteomic data associated with observed physiological parameters under cold stress were the remarkable changes in two types of proteins: peroxidases and aquaporins. Here we focus intensively on these two proteins, and some of the important pathways and possible mechanisms associated with the observed cold tolerance in Dajiao.

### Peroxidases in Response to Cold Stress

The genes encoding plant peroxidases are part of a large multi-gene family of 73, 138, and approximately 200 members in *Arabidopsis*, rice, and maize, respectively. Thus, it is generally difficult to define the exact functions of each peroxidase owing to the presence of so many isozymes with low substrate specificity (Hiraga et al., 2001). Proteomics analysis has an advantage in uniquely identifying some of the peroxidase isoforms, offering a promising approach for dissecting mechanisms of individual peroxidases attributed to cold tolerance at the global level. In our study, ROS accumulation and wilting were physiological and biochemical phenotypes of Cavendish banana, but not Dajiao, under cold treatment (**Figures 2A–M**). Corresponding to this, our proteomic data showed that four peroxidases (Peroxidase 52, Peroxidase 15, Peroxidase 59, and Peroxidase P7) located in membranes were differentially abundant, and all showed an increased abundance at either 3 or 6 h in Cavendish or Dajiao (**Supplementary Table S2**). Further analysis showed that Peroxidase 59 was increased only in Cavendish after 6 h of cold treatment, Peroxidase 15 was increased in both Cavendish and Dajiao at 6 h of cold treatment, while Peroxidase 52 and Peroxidase P7 were increased only in Dajiao at 3 h of cold treatment. Our data indicate that Peroxidase 52 and Peroxidase P7 responded to the cold treatment considerably faster in Dajiao than in Cavendish. These important proteomics results were cross-verified by multiple technologies. For example, our RT-PCR data suggest that *peroxidase P7* and *peroxidase 52* are induced earlier in Dajiao (**Figures 6F,H**). The expression changes of Peroxidase 59 and Peroxidase P7 were also verified by MRM analysis (**Figure 6J**), further demonstrating that Peroxidase P7 is cold-induced in Dajiao but not in Cavendish. In addition, we used a biochemistry approach to determine that peroxidase-mediated ROS scavenging is associated with cold tolerance of Dajiao, after Cavendish and Dajiao seedlings were treated with a peroxidase inhibitor (NaN_3_). We found that the peroxidase inhibitor impaired the capacity of ROS scavenging and substantially compromised the cold tolerance of Dajiao (**Figure 7**). Our data support the notion that Dajiao has more cold tolerance than Cavendish to some extent due to the rapid increase of membrane-bound Peroxidase 52 and Peroxidase P7, which are involved in membrane protective functions by detoxifying H_2_O_2_ at an early stage of cold stress.

In our previous study on the responses of Dajiao to cold stress using quantitative proteomics analysis, five peroxidases were differentially expressed. Four of them are located in the cytoplasm, being down-regulated under cold stress, while one (Peroxidase 52) is located in membrane, the only peroxidase with increased abundance (Yang et al., 2012). In the current membrane proteomics analysis, we found that both membrane peroxidases (Peroxidase P7 and Peroxidase 52) showed increased abundance in Dajiao. Combining the two proteomics data shows that differentially abundant peroxidases located in membranes are increased in Dajiao under cold stress. We suspect that the enhanced cold tolerance of Dajiao might be associated with more robust detoxification of ROS by increased abundance of membrane peroxidases. It is conceivable that some peroxidases are extractable and soluble at low ionic strength, and some are ionically bound to the cell-wall matrix, requiring high ionic strength solvent for efficient extraction. The class III peroxidases are mainly found in the cell walls and vacuoles (Passardi et al., 2004). Zhang et al. (2011) showed that higher activities of this type of peroxidases to a certain extent could be used to explain the higher cold tolerance of Dajiao, but ionically bound CWPs were not involved. To gain insight into the peroxidative function, both ionically bound CWP (**Figure 8B**) and soluble peroxidase (**Figure 8A**) activities were determined in Cavendish and Dajiao under 10°C treatment for 0, 3, 6, 24, and 48 h. As shown in **Figure 8A**, we found that the cold-sensitive Cavendish had a higher induction rate of soluble peroxidase activity than did the cold-tolerant Dajiao, which showed a discreet increase in peroxidase activity at an early stage and reduction of this activity during extended cold stress. Similar results were reported by Dou et al. (2016), and a similar phenomenon was found in response to other abiotic stresses (Hosseini et al., 2007). Strikingly, the ionically bound CWP activity showed a quite different pattern compared with the soluble peroxidase activity, as shown in **Figure 8B**. In Cavendish, the ionically bound CWP activity was induced at 6 h but remained at a lower level at 24 and 48 h than at 0 h. However, levels of the enzyme activity in Dajiao were rapidly increased at 3 h and remained stable until a second significant increase at 48 h. As catalases are mainly localized in peroxisomes, the ionically bound CWPs are the primary H_2_O_2_-scavenging enzymes that detoxify H_2_O_2_ in the apoplast of the plant cells. Combined with the ROS accumulation in Dajiao after the cold treatment (**Figures 2J–M**), we believe that the fast increase in and sustained activity of ionically bound CWP in Dajiao is likely to play a crucial role in the detoxification of H_2_O_2_ in the leaves. It should be noted that a slight wilt phenotype and a significant increase in ionically bound CWP activity occurred simultaneously in Dajiao at 48 h of cold treatment (data not shown). This suggests that ionically bound CWPs may be induced by cold stress.

H_2_O_2_ can be induced by almost all forms of abiotic and biotic stress; it is known as an oxidant and functions as a signal molecule in plants, as proved by innumerous studies (Gill and Tuteja, 2010). Thus, plants need to rigorously control the level of apoplastic H_2_O_2_. Taken together, the iTRAQ data and enzyme activity results lead us to hypothesize that effectively regulating the balance of the oxidative burst and detoxification of H_2_O_2_ by a rapid increase in the abundance of membrane-bound Peroxidase 52 and Peroxidase P7, and ionically bound CWP activity may at least in part explain the greater tolerance of Dajiao than Cavendish to cold stress. Our preliminary phosphoproteomics analysis showed that peroxidase activity was also selectively regulated by phosphorylation, which affects the cold resistance of Dajiao to a certain extent (data not shown).

### Change of Aquaporins Under Cold Stress

As most abiotic stresses result in cellular dehydration, the ability to maintain water balance in cells via increasing water use efficiency and the promotion of plant water retention to sustain cellular structure and function, seems critically important for plants to improve tolerance to abiotic stress (Mahdieh et al., 2008; Shareena et al., 2013). Water bidirectional movement across cellular membranes is regulated largely by aquaporins, a type of MP belonging to the MIP (Membrane Intrinsic Proteins) family. These aquaporins are mainly divided into seven subfamilies depending on membrane localization and amino acid sequence, including plasma membrane intrinsic proteins (PIPs) and tonoplast intrinsic proteins (TIPs) (Chaumont et al., 2001), which are the two main aquaporins stimulated by various stresses. The cellular and tissue hydraulic function of each aquaporin isoform has been studied using reverse genetics and over expression during the last decade, and genome-wide comparative analysis of aquaporin genes has also been conducted in recent years (Hu et al., 2015). To date, few proteomics analyses on aquaporins have been reported. Our data showed that four PIPs (MaPIP1;1, MaPIP1;2, MaPIP2;4, and MaPIP2;6) and two TIPs (MaTIP1;1 and MaTIP1;3) were differentially abundant in Cavendish or Dajiao (**Supplementary Table S1**). Among them, MaPIP1;1, MaPIP2;6, and MaTIP1;3 were increased in Cavendish at 6 h of cold treatment, and four aquaporins were increased in abundance at the early stage of 3 h of cold treatment in Dajiao. There was no difference in terms of the number of differentially increased aquaporins; however, increased abundance of aquaporins at an earlier stage of cold stress was observed in Dajiao but not in Cavendish, which is similar to results found for peroxidases. We measured the transcriptional level of *MaPIP1;1, MaPIP1;2, MaPIP2;6a, MaPIP2;6b*, and *MaTIP1;3* in both Cavendish and Dajiao treated for 0, 3, 6, 24, and 48 h at 10°C (**Figures 6A–E**), and the protein expression of MaPIP2;4 by MRM (**Figure 6J**). The validation data also confirmed that our discovery-based membrane proteomics and data on aquaporins are reliable.

Previous studies on gene overexpression and suppression indicated that *PIP1;1* plays a very important role in improvement of plant tolerance to abiotic stress by decreasing water loss, increasing root biomass, or increasing root hydraulic conductance (Tsuchihira, 2010). Heterologous expression of banana *MaPIP1;1* in *Arabidopsis* was reported to confer tolerance to salt and drought stress, and it was proposed that this transgenic species may reduce membrane injury, improve ion distribution, and maintain osmotic balance (Xu et al., 2014). *MaPIP2;6*-overexpressing banana plants displayed higher photosynthetic efficiency and decreased membrane damage under salt stress conditions (Sreedharan et al., 2015). Both *MaPIP1;1* and *MaPIP2;6* function to maintain plant water potential and reduce membrane damage. Interestingly, our proteomics data showed that Dajiao responded with an increased abundance of the two aquaporins at 3 h of cold stress, but Cavendish did not. Furthermore, Shareena et al. (2013) demonstrated that transgenic lines overexpressing *MaPIP1;2* had lower MDA level, elevated proline and relative water content, and higher photosynthetic efficiency as compared with equivalent controls under different abiotic stress conditions. Our proteomics data showed that MaPIP1;2 was decreased at 6 h of cold treatment in Cavendish, but no change was observed in Dajiao (**Supplementary Table S1**), which is consistent with the previous reports. Thus, we suspect at least three early responding aquaporins (MaPIP1;1, MaPIP1;2, and MaPIP2;6) are likely to be associated with the cold resistance of Dajiao.

We further investigated the possible role of six differentially abundant aquaporins in maintaining leaf water content, by assessment of the phylogenetic relationship with two model plant species based on alignment of the aquaporin sequences. The resulting phylogenetic tree (**Supplementary Figure S7**) showed that *MaPIP1;1, MaPIP1;2, MaPIP2;4, MaPIP2;6, MaTIP1;1*, and *MaTIP1;3* are most closely related to *OsPIP1;1, OsPIP1;3, OsPIP2;3, OsPIP2;6*, and *OsTIP1;1* from rice and *AtTIP1;1* from *Arabidopsis*, respectively. *OsPIP1s* and *OsPIP2;1* were reported to be mainly located in mesophyll cells, with minor presence in the bundle sheath and epidermis (Sakurai et al., 2008). Additionally, *AtPIP2;1* is expressed predominantly in the veins, while *AtTIP1;1* and *AtPIP2;6* are exclusively present in the veins. Since ABA enables alteration of leaf hydraulic conductance and water permeability of bundle sheath protoplasts but not mesophyll protoplasts, it has been suggested that the bundle sheath may create a significant hydraulic barrier in *Arabidopsis thaliana* leaves (Shatil-Cohen et al., 2011). It is of note that the TIPs control cell water and osmotic potential homeostasis through fast water exchanges between the vacuole and the cytoplasm. Thus, different roles in the maintenance of water potential in leaves are anticipated for mesophyll and bundle sheath cell-specific aquaporins. It is conceivable that the big difference in phenotypic and physiological responses to cold stress between Cavendish and Dajiao may help us better understand the role of aquaporins. The aquaporin results in this study support the observed phenotype that the leaves of Cavendish quickly wilted but no difference was observed in Dajiao under cold treatment. Meanwhile, we found that there was no difference in stomatal conductance and transpiration rate between Cavendish and Dajiao (Gao et al., 2017). Since water status of plant leaves is dependent on both stomatal regulation and water supply from the vasculature to inner tissues, water appears to be supplied more effectively from the vasculature in Dajiao than in Cavendish. Taking these considerations into account, we suspect that the faster and earlier responding MaPIP1;1, MaPIP2;4, MaPIP2;6, and MaTIP1;3 and the stable expression of MaPIP1;2 in the leaf veins may improve water diffusion out of the veins. Expression of MaPIP1;1, MaPIP1;2, and MaPIP2;4 in mesophyll cells to maintain water potential may represent one important role in Dajiao’s cold tolerance. These speculations need to be verified.

### Other Pathways in Response to Cold Stress

Sensing low temperature can signal either an acclimation response for reducing chilling injury or one leading to the deterioration of the tissue, and cellular membranes are probably one of the sensors (Lukatkin et al., 2012). Saturated fatty acids generate more rigid membranes, and they are associated with chilling sensitivity, while unsaturated fatty acids, creating fluid membranes, are associated with lower sensitivity to cold. Manipulating the expression of single genes involved in lipid metabolism can reduce plants’ sensitivity to cold temperatures (Sui et al., 2007). A significantly increased MDA content (**Figure 2N**) and cell membrane permeability (**Figure 2O**) in Cavendish, with no equivalent changes in Dajiao, suggests that cold treatment appears to result in damage to membrane lipids of Cavendish, while Dajiao is likely to maintain the integrity of membranes. An increased *GDPD* was reported to be involved in photooxidative-dependent signaling pathways, in the cold-resistant wild type of barley (*Hordeum vulgare*) under cold treatment (Svensson et al., 2006). Our proteomics data showed that abundance of two GDPDs was increased in Dajiao but no changes were observed in Cavendish under cold treatment for 3 h, and their gene expression patterns were consistently in line with the proteomics data (**Supplementary Figure S6**). Additionally, the function of transporters located in membranes may also be affected by damage to lipid membranes caused by cold treatment. The ABC transporter A family member 7 was not detected in Cavendish but profoundly increased in Dajiao at 3 and 6 h (**Supplementary Figure S6I**). RT-PCR data also showed that the *ABC transporter A family member 7* was up-regulated in Dajiao at 3 and 6 h, but no changes were observed in Cavendish (**Supplementary Figure S6E**). The improvement of soluble sugars after low-temperature treatment is one of the other important biochemical indexes of plant cold resistance. A comparison study between cold-sensitive Cavendish and Prata, a more cold-resistant cultivar, revealed higher levels of enzymes involved in starch metabolism (amylase β) and sucrose increase (sucrose phosphate synthase) in the resistant cultivar (Agopian et al., 2011). In our proteomics data, we found that two glucan endo-1,3-beta-glucosidases were induced in Dajiao but no changes were detected in Cavendish (**Supplementary Figure S6I**). Similarly, *glucan endo-1,3-beta-glucosidases 8a* was up-regulated at 3 h and *glucan endo-1,3-beta-glucosidases 8b* was up-regulated at 6 h in Dajiao but both were down-regulated in Cavendish (**Supplementary Figures S6A,B**). These findings suggest that soluble sugar and its metabolism appear to be involved in the cold adaptation of Dajiao.

Taken together, our data provide new insights into the following regulatory mechanisms contributing to the greater tolerance of Dajiao than Cavendish to cold stress. First, rapid increase in abundance of membrane-bound Peroxidase 52, Peroxidase P7, and ionically bound CWP activity at an early stage of cold stress allowed control of H_2_O_2_ concentrations by precisely regulating the balance of the oxidative burst and detoxification, and thus minimizing ROS-induced damage. Second, early increased abundance of MaPIP1;1, MaPIP2;4, MaPIP2;6, and MaTIP1;3 and stable expression of MaPIP1;2 in the leaf veins in response to cold stress seems to facilitate water diffusion out of the veins, and expression of MaPIP1;1, MaPIP1;2, and MaPIP2;4 in mesophyll cells allows maintenance of leaf cell water potential and functions. Finally, MPs involved in lipid metabolism and sugar metabolism are beneficial for improving Dajiao membrane stability and regulating osmotic potential.

## Conclusion

In this study, we implemented an effective method by combining iTRAQ-based membrane proteomics analysis with the use of transmembrane structure prediction software to identify candidate MPs differentially regulated in response to cold stress toward understanding the plausible mechanism of cold tolerance. We found two groups of candidate proteins that were consistently induced at an early stage of cold stress and appeared responsible for Dajiao’s high cold tolerance. The first family of proteins containing membrane peroxidases is involved in the H_2_O_2_ scavenging system and ROS signaling networks. The second family of proteins involves aquaporins that function in promoting water diffusion out of the veins as well as maintaining leaf cell water potential and functions. To the best of our knowledge, this is the first report using quantitative proteomic analysis for global MP responses to cold stress in two contrasting banana genotypes. The protocols reported here can be used for other plant membrane proteomics analyses of the entire cell membrane tissue.

## Availability of Data and Materials

All the data supporting these findings are contained within the manuscript and supplementary files.

## Author Contributions

Q-SY, G-JY, JG, J-HL, and W-DH conceived and designed the study, and wrote the manuscript. T-XD, X-HS, F-CB, OS, G-MD, and C-HH cultivated the banana plantlets, extracted RNA and detected enzyme activity, and participated in the bioinformatics analysis. SZ, X-HS, and C-YL drafted the manuscript, designed the tables, critically reviewed the manuscript, and provided the guidance. All authors read and approved the final manuscript.

## Conflict of Interest Statement

The authors declare that the research was conducted in the absence of any commercial or financial relationships that could be construed as a potential conflict of interest.

## Supplementary Material

FIGURE S1 Venn diagrams of results for all protein IDs identified in three sets of biological replicate samples from Cavendish **(A)** and Dajiao **(B)**.

FIGURE S2 Venn diagrams of results for all protein IDs with quantification ratios in three sets of biological replicate samples from Cavendish **(A)** and Dajiao **(B)**.

FIGURE S3 Comparison of log_2_ iTRAQ ratio (115/114 and 116/114) for 2,333 proteins identified in the three Cavendish biological replicates, set1, set2, and set3. Plots of 115/114 or 116/114 ratios for each of the quantified proteins between two of the three sets generated comparable quantification results, as determined by a linear regression analysis that revealed a slope of around 0.88, 0.87, or 0.87 in Cavendish, among the pairs of set1/set2 **(A)**, set1/set3 **(B)**, and set2/set3 **(C)**, respectively. Similar plots were also conducted for the 116/114 ratio, yielding a slope of around 0.88, 0.87, or 0.89 in Cavendish, among the pairs of set1/set2 **(D)**, set1/set3 **(E)**, and set2/set3 **(F)**, respectively.

FIGURE S4 Comparison of GO biological processes for Cavendish and Dajiao MPs **(A)** and DAMPs **(B)** under cold stress.

FIGURE S5 Comparison of pathway enrichment analysis for Cavendish and Dajiao MPs **(A)** and DAMPs **(B)** under cold stress.

FIGURE S6 Relative mRNA levels in Cavendish and Dajiao seedlings were determined by quantitative RT-PCR analyses. Relative expression of two glucan endo-1,3-beta-glucosidases 8 **(A,B)** and two GDPDs **(C,D)**, ABC transporter A family member 7 **(E)**, Aspartic proteinase **(F)**, Nicotiana lesion-inducing **(G)**, Inactive receptor kinases At2g26730 **(H)** in Cavendish and Dajiao under 10°C for 0, 3, 6, 24, and 48 h, and their expression patterns in proteomics data **(I)**. The horizontal arrow, upwardly tilted arrow, and downwardly tilted arrow represent no change, and increased and decreased expression, respectively.

FIGURE S7 Phylogenetic tree of aquaporins from *Musa acuminate*.

TABLE S1 Differentially abundant aquaporins with iTRAQ ratios ≥ or ≤ 1.4-fold.

TABLE S2 Differentially abundant peroxidases with iTRAQ ratios ≥ or ≤ 1.4-fold.

DATA S1 Contains nine spreadsheets: The first spreadsheet contains explanations of column headings and sheet names; Cavendish set1 shows the list of identified proteins from the first biological replicate of Cavendish banana samples at 10°C for 0, 3, and 6 h; Cavendish set2 shows the list of identified proteins from the second biological replicate of Cavendish banana samples at 10°C for 0, 3, and 6 h; Cavendish set3 shows the list of identified proteins from the third biological replicate of Cavendish banana samples at 10°C for 0, 3, and 6 h; Dajiao set1 shows the list of identified proteins from the first biological replicate of Dajiao banana samples at 10°C for 0, 3, and 6 h; Dajiao set2 shows the list of identified proteins from the second biological replicate of Dajiao banana samples at 10°C for 0, 3, and 6 h; Dajiao set3 shows the list of identified proteins from the third biological replicate of Dajiao banana samples at 10°C for 0, 3, and 6 h; Cavendish MPs shows the list of Cavendish membrane proteins predicted by at least two of the three web programs Phobius, Scampi-single, and TMHMM; Cavendish DAMPs shows the list of Cavendish differentially abundant membrane proteins with iTRAQ ratios ≥ or ≤ 1.4-fold; Dajiao MPs shows the list of Dajiao membrane proteins predicted by at least two of the three web programs Phobius, Scampi-single, and TMHMM; Dajiao DAMPs shows the list of Dajiao differentially abundant membrane proteins with iTRAQ ratios ≥ or ≤ 1.4-fold.

DATA S2 Primers for RT-PCR and subcellular localization.

## Abbreviations

ABA: abscisic acid
CAT: catalase
CWP: cell-wall peroxidase
DAMP: differentially abundant membrane protein
DDA: data-dependent acquisition
FDR: false discovery rate
GDPD: glycerophosphoryl diester phosphodiesterase
GFP: green fluorescent protein
GO: gene ontology
HCD: high energy collision dissociation
hpRP: high pH reverse phase
iTRAQ: isobaric tags for relative and absolute quantitation
LC: liquid chromatography
MAPK: mitogen-activated protein kinase
MDA: malondialdehyde
MMTS: methyl-methyl-thiomethyl sulfoxide
MP: membrane protein
MRM: multiple reaction monitoring
MS: mass spectrometry
POD: peroxidase
ROS: reactive oxygen species
RSD: relative standard deviation
RT-PCR: reverse-transcription PCR
TCEP: tris-(2-carboxyethyl) phosphine
