# Supplementary figures and images for: Early Cold-Induced Peroxidases and Aquaporins Are Associated With High Cold Tolerance in Dajiao (Musa spp. ‘Dajiao’)
Source: Front Plant Sci. 2018 Mar 8;9:282. doi: 10.3389/fpls.2018.00282 (PMC5852111; doi:10.3389/fpls.2018.00282)

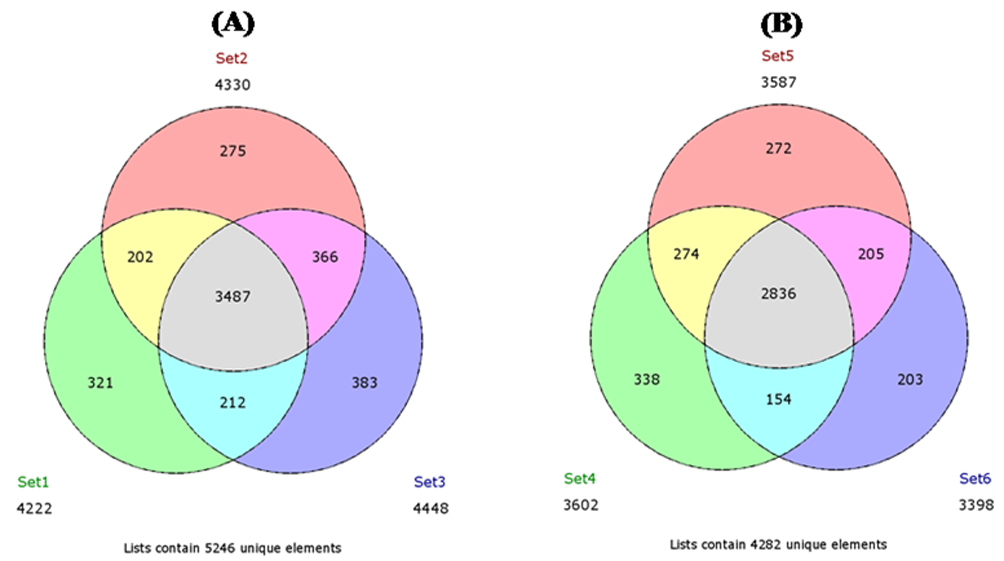

Supplement: FIGURE S1 — Venn diagrams of results for all protein IDs identified in three sets of biological replicate samples from Cavendish (A) and Dajiao (B). [file Image_1.TIF]

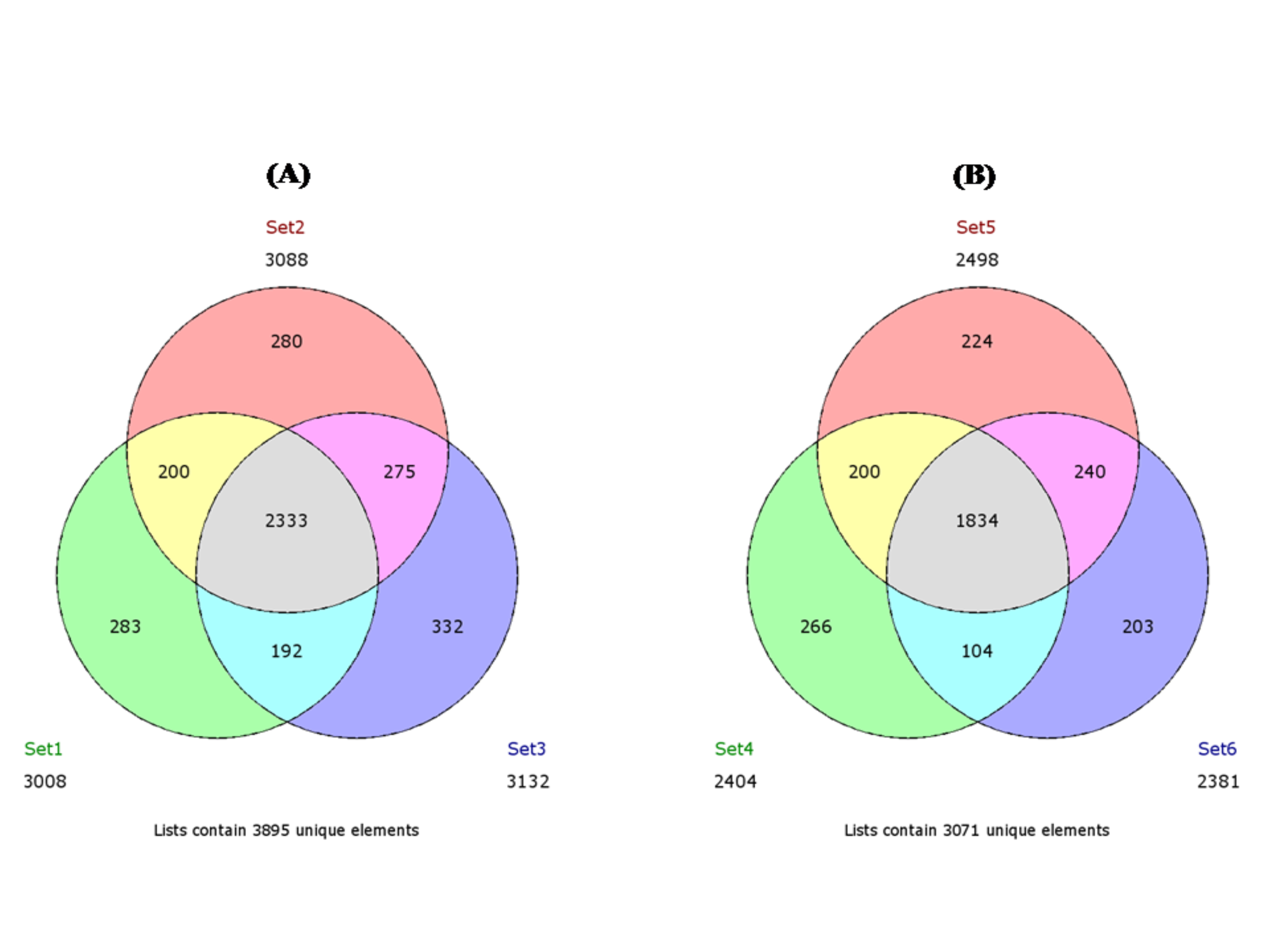

Supplement: FIGURE S2 — Venn diagrams of results for all protein IDs with quantification ratios in three sets of biological replicate samples from Cavendish (A) and Dajiao (B). [file Image_2.TIFF]

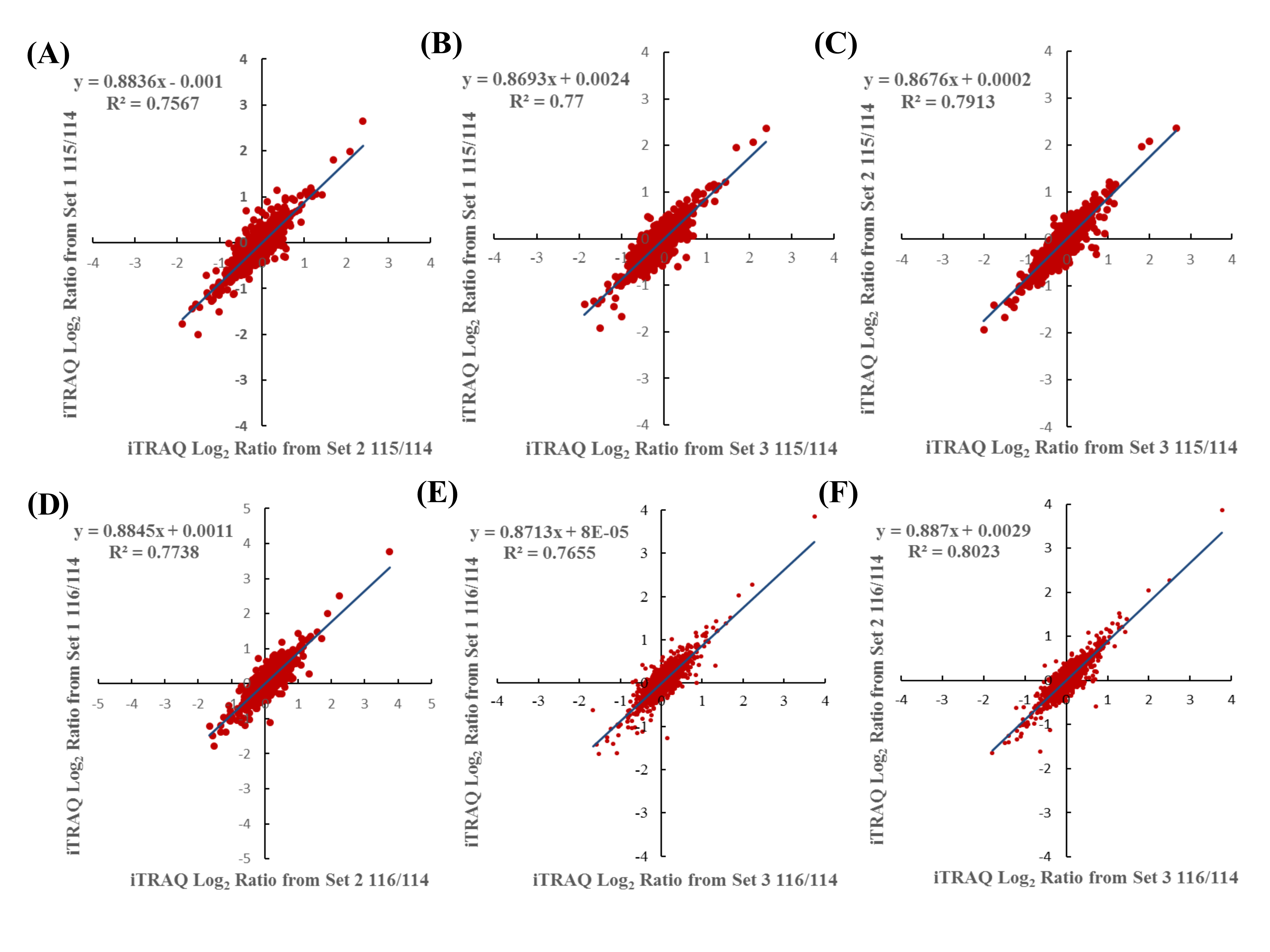

Supplement: FIGURE S3 — Comparison of log2 iTRAQ ratio (115/114 and 116/114) for 2,333 proteins identified in the three Cavendish biological replicates, set1, set2, and set3. Plots of 115/114 or 116/114 ratios for each of the quantified proteins between two of the three sets generated comparable quantification results, as determined by a linear regression analysis that revealed a slope of around 0.88, 0.87, or 0.87 in Cavendish, among the pairs of set1/set2 (A), set1/set3 (B), and set2/set3 (C), respectively. Similar plots were also conducted for the 116/114 ratio, yielding a slope of around 0.88, 0.87, or 0.89 in Cavendish, among the pairs of set1/set2 (D), set1/set3 (E), and set2/set3 (F), respectively. [file Image_3.TIFF]

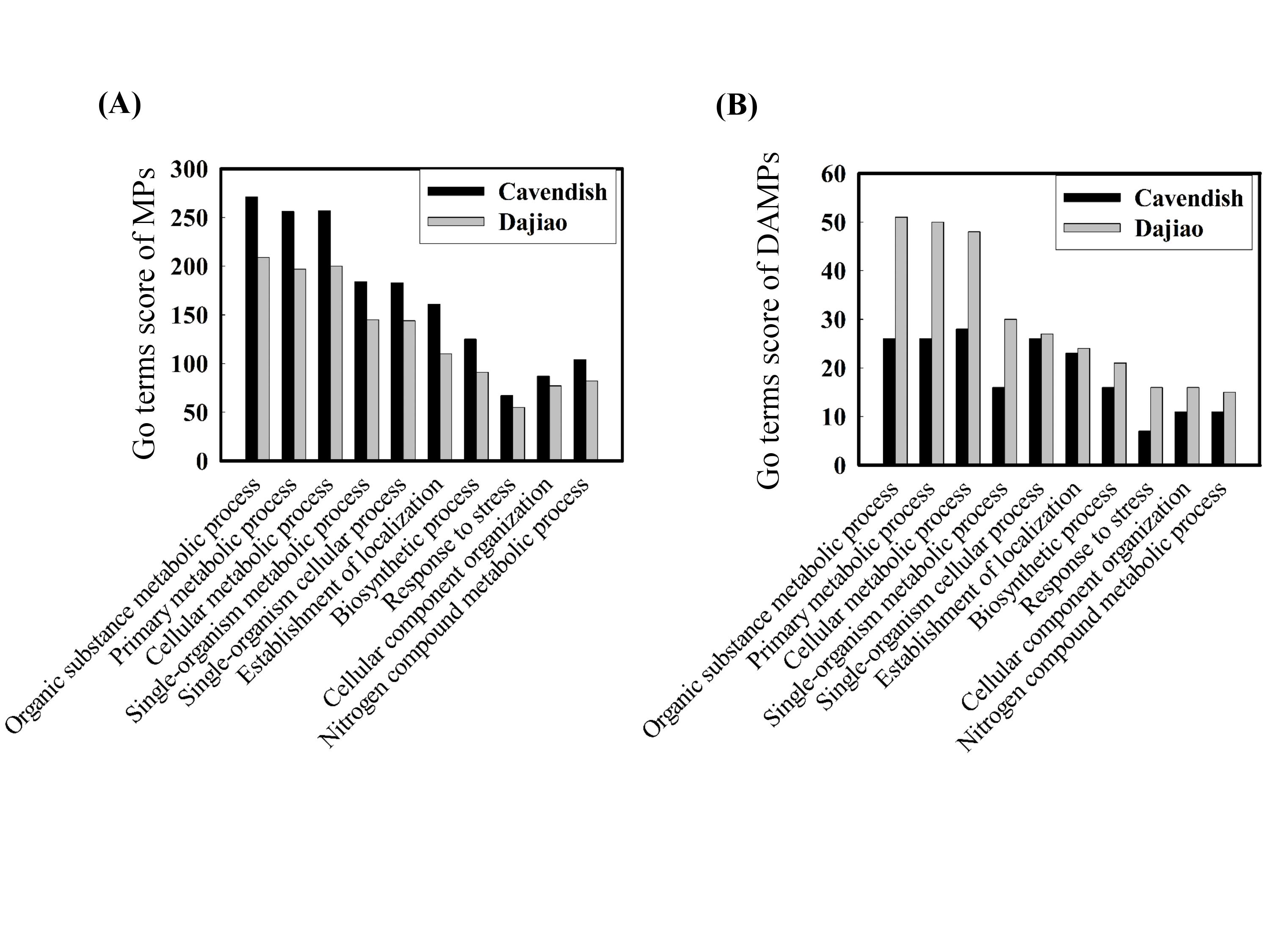

Supplement: FIGURE S4 — Comparison of GO biological processes for Cavendish and Dajiao MPs (A) and DAMPs (B) under cold stress. [file Image_4.JPEG]

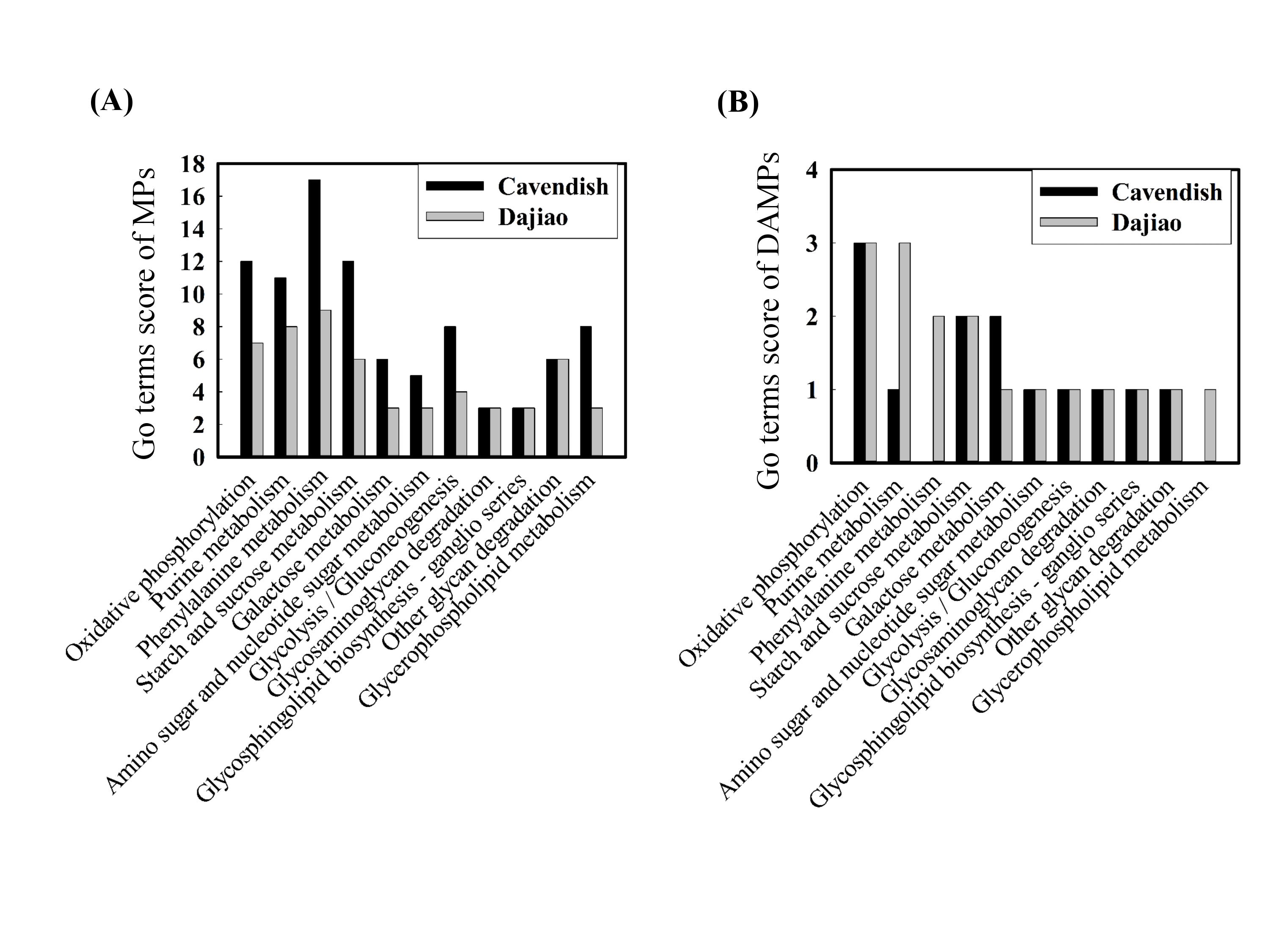

Supplement: FIGURE S5 — Comparison of pathway enrichment analysis for Cavendish and Dajiao MPs (A) and DAMPs (B) under cold stress. [file Image_5.JPEG]

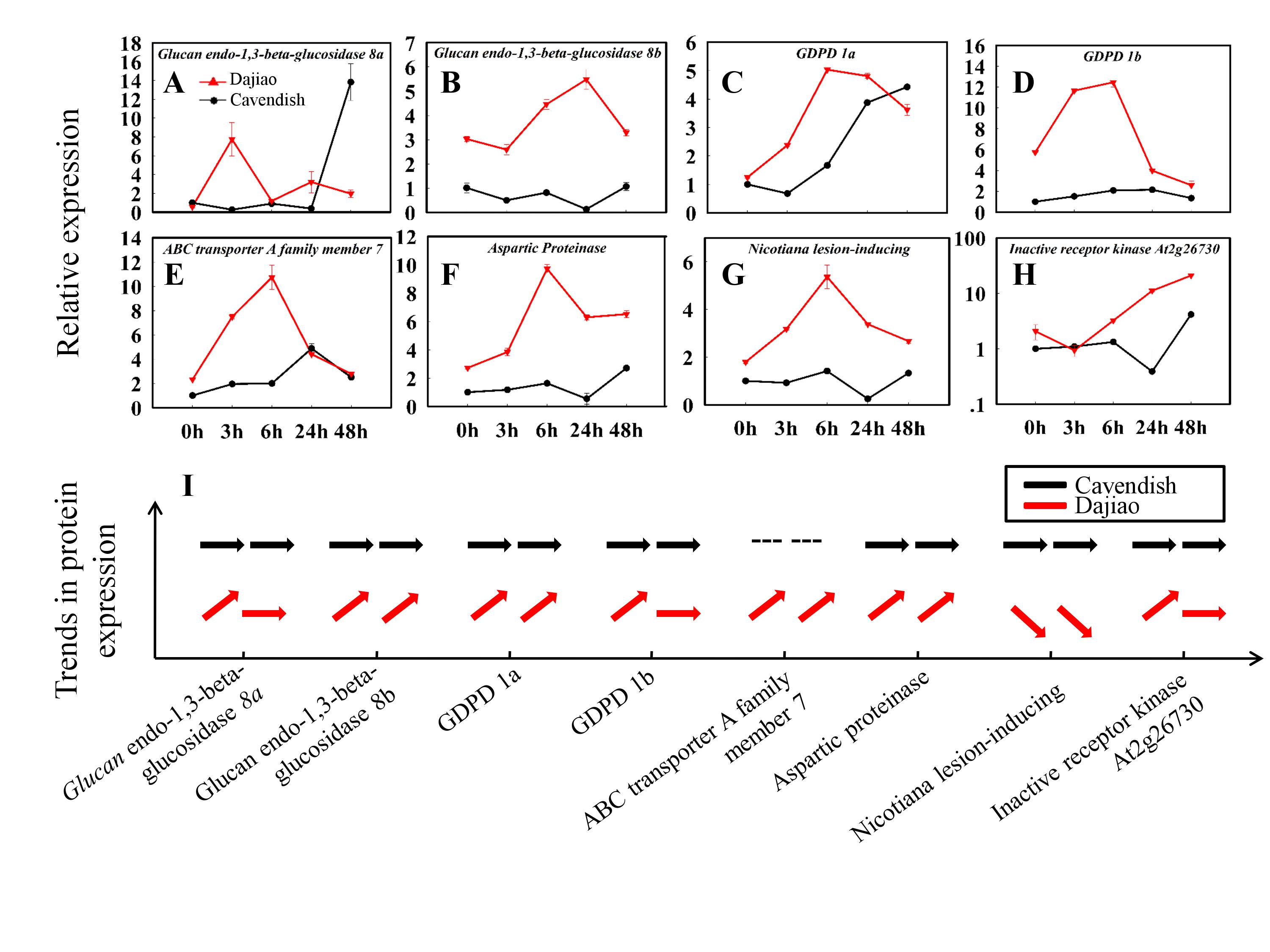

Supplement: FIGURE S6 — Relative mRNA levels in Cavendish and Dajiao seedlings were determined by quantitative RT-PCR analyses. Relative expression of two glucan endo-1,3-beta-glucosidases 8 (A,B) and two GDPDs (C,D), ABC transporter A family member 7 (E), Aspartic proteinase (F), Nicotiana lesion-inducing (G), Inactive receptor kinases At2g26730 (H) in Cavendish and Dajiao under 10°C for 0, 3, 6, 24, and 48 h, and their expression patterns in proteomics data (I). The horizontal arrow, upwardly tilted arrow, and downwardly tilted arrow represent no change, and increased and decreased expression, respectively. [file Image_6.JPEG]

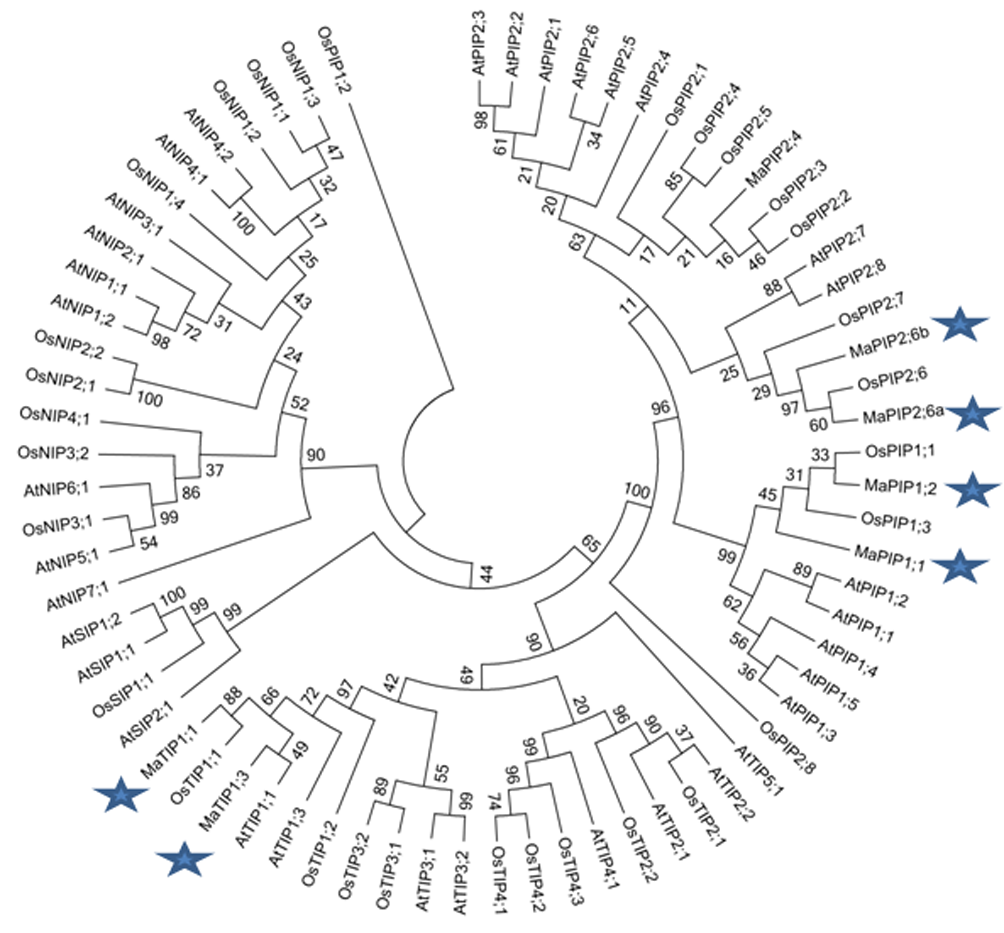

Supplement: FIGURE S7 — Phylogenetic tree of aquaporins from Musa acuminate. [file Image_7.TIF]
