# Supplementary material for: Early Cold-Induced Peroxidases and Aquaporins Are Associated With High Cold Tolerance in Dajiao (Musa spp. ‘Dajiao’)
Source: Front Plant Sci. 2018 Mar 8;9:282. doi: 10.3389/fpls.2018.00282 (PMC5852111; doi:10.3389/fpls.2018.00282)
Supplement: TABLE S1 — Differentially abundant aquaporins with iTRAQ ratios ≥ or ≤ 1.4-fold. [file Table_1.DOCX]

**Table S1. Differentially** **abundant aquaporins with iTRAQ ratios ≥ or ≤ 1.4-fold.**

| Protein id | Protein name | Cavendish | | | Dajiao | | | | |
| --- | --- | --- | --- | --- | --- | --- | --- | --- | --- |
|  |  | Trends ^a^ | 3h | 6h | | Trends ^a^ | 3h | 6h |  |
| Ma04_p21270 | Aquaporin PIP1-1 | = + | 0.948 ± 0.040 | 1.565 ± 0.150 | | + = | 1.478 ± 0.060 | 0.993 ± 0.034 |  |
| Ma04_p20470 | Aquaporin TIP1-1 | = = | 0.781 ± 0.021 | 0.998 ± 0.048 | | =－ | 1.071 ± 0.105 | 0.671 ± 0.043 |  |
| Ma04_p22160 | Aquaporin PIP2-6a | = + | 0.749 ± 0.096 | 1.519 ± 0.086 | | = = | 1.115 ± 0.103 | 0.943 ± 0.111 |  |
| Ma02_p13080 | Aquaporin PIP2-6b | = + | 0.888 ± 0.055 | 1.465 ± 0.120 | | + = | 1.431 ± 0.142 | 0.949 ± 0.068 |  |
| Ma06_p16930 | Aquaporin TIP1-3 | = + | 1.115 ± 0.040 | 1.648 ± 0.087 | | + + | 1.640 ± 0.028 | 1.402 ± 0.018 |  |
| Ma03_p14410 | Aquaporin PIP1-2 | =－ | 1.119 ± 0.069 | 0.616 ± 0.086 | | = = | 1.235 ± 0.012 | 0.959 ± 0.027 |  |
| Ma02_p00190 | Aquaporin PIP2-4 | --- | --- | --- | | + = | 1.449 ± 0.005 | 1.311 ± 0.022 |  |

^a^+, up-regulated; －, down-regulated; =, not significantly changed; ---, not detected.

**Table S2. Differentially abundant peroxidases with iTRAQ ratios ≥ or ≤ 1.4-fold.**

| Protein id | Protein name | Cavendish | | | Dajiao | | | |
| --- | --- | --- | --- | --- | --- | --- | --- | --- |
|  |  | Trends ^a^ | 3h | 6h | Trends ^a^ | 3h | 6h |  |
| Ma10_p25840 | Peroxidase 15 | = + | 1.248 ± 0.051 | 1.581 ± 0.057 | = + | 1.239 ± 0.041 | 1.980 ± 0.023 |  |
| Ma10_p27800 | Peroxidase 7 | = = | 1.334 ± 0.087 | 0.909 ± 0.029 | + = | 1.414 ± 0.195 | 1.213 ± 0.097 |  |
| Ma02_p17270 | Peroxidase 52 | = = | 0.977 ± 0.082 | 0.978 ± 0.105 | + = | 1.668 ± 0.133 | 1.093 ± 0.049 |  |
| Ma02_p23580 | Peroxidase 59 | = + | 1.209 ± 0.059 | 1.636 ± 0.100 | --- | --- | --- |  |

^a^+, up-regulated; =, not significantly changed; ---, not detected.
